# A set of guidelines as support for the integrated geo-environmental characterization of highly contaminated coastal sites

**DOI:** 10.1038/s41598-024-58686-4

**Published:** 2024-04-08

**Authors:** Angela Rizzo, Giovanni Scicchitano, Giuseppe Mastronuzzi

**Affiliations:** 1 Department of Earth and Geo-Environmental Sciences, University of Bari Aldo Moro, Via Orabona, 4, 70125 Bari, Italy; 2 Interdepartmental Research Centre for Coastal Dynamics, University of Bari Aldo Moro, Via Orabona, 4, 70125 Bari, Italy

**Keywords:** Geological model, Anthropogenic impacts, Morphodynamic variations, Climate change, Apulia region, Environmental impact, Climate-change impacts

## Abstract

The knowledge of geomorphodynamic aspects is crucial for understanding marine and coastal processes/dynamics as well as for characterizing coastal environments heavily affected by anthropogenic activities. To provide a framework of analysis that can be applied in a consistent way for the geo-environmental characterization of highly contaminated coastal sites, in this paper a set of operational guidelines is proposed. Special attention is given to the role of geomorphological-based surveys and analyses in defining (i) the site-specific geological model of the investigated site, (ii) the anthropogenic impacts on marine and coastal sediments, (iii) the expected morphodynamic variations induced by climate change and anthropogenic interventions, (iv) tailored dissemination activities and community engagement plans. Then, an evaluation of the state of the art of activities already performed for the characterization of the coastal contaminated sites located in the Apulia region (southern Italy) is provided. The outcomes of this research are also provided in the form of infographics to favor their dissemination among communities and stakeholders.

## Introduction

To date, about 40% of the worldwide population lives within 100 km from the coasts (UNEP, 2017), which results in having a population density value twice the global one. Due to their favorable climatic and environmental condition, coastal areas have attracted human who placed their settlements and activities since historical times. On the other hand, coastal zones host a variety of marine and terrestrial ecosystems, which provide a number of ecosystem services (ES), which contribute to human well-being^1^. As a consequence of the high urbanization and industrialization trends and the increasing resource demand, currently, coastal zones, and in particular estuaries and sheltered areas, represent highly altered environments^2,3^.

In this context, the European Environment Agency (EEA) has highlighted that the Mediterranean Sea is characterized by intense human activities that are causing strong environmental impacts in the form of coastal and marine degradation^4^ (EEA, 2001). Principal driving forces and pressures are represented by urbanization, tourism, agriculture, fisheries, aquaculture, industry, and maritime transport. As far as the industrial sector is concerned, it emerged that a large range of different industrial plants (from mining to manufactured products) are located around the Mediterranean basin with hot-spot areas mainly in the north-west sector, where heavy industry plans and remarkable commercial harbors are hosted. Pressures from heavy industrial plants in the Mediterranean area include also chemical, petrochemical, and metallurgy sectors. By way of example, the biggest steel plant in Europe is located in southern east Italy, along the Ionian Sea. Industrial activities may induce direct and indirect impacts on the coastal areas and related ecosystems. Direct impacts are largely related to the widespread of contaminants (organic and inorganic) in all the different marine and coastal matrices, as they can be dissolved in water, stored in sediments, or accumulated in biota. Indirect impacts include the modification of geomorphodynamic processes as well as the changes in the structures of the coastal and seabed habitat and morphologies, affecting the seafloor integrity and coastal dynamics^5^.

Contaminated sites are those areas in which, due to past or ongoing anthropogenic activities, environmental matrices resulted to be directly and indirectly affected by pollution. The Italian coastal sector hosts a high number of contaminated sites, 17 of them included in the list of Sites of National Interest (“SIN” in Italian). According to the Italian legislature (Legislative Decree n. 152/2006), SINs represent “*wide portions of the national territory (including any surface water bodies and their sediments) with remarkable environmental value identified by the national law based on their characteristics that entail a high health and ecological risk due to the density of the population or the extent of the site itself, as well as a significant socio-economic impact and risk to historical and cultural interest sites*.” Based on the most recent perimeter delimitation, about 77,000 hectares of marine and coastal areas are included in the SIN boundaries (https://www.isprambiente.gov.it/it/attivita/suolo-e-territorio/siti-contaminati/siti-di-interesse-nazionale-sin).

The environmental characterization of the marine areas in SINs has been performed by the Italian Institute for Environmental Research and Application (ISPRA) funded by the Italian Environmental Ministry. The proposed investigation strategy was based on a specific procedure defined taking into account the main European Environmental legislations (e.g., Water Framework Directive, 2000/60/EC, OSPAR, 1992) and it was focused on the analysis of contaminants’ distribution in the marine matrices (sediments, water, and biota). Special attention was given to the sediment characterization, by defining sampling strategy and physical, chemical, and ecotoxicological analyses^6^. Similarly, the characterization programs carried out by the Regional Agencies for Environmental Protection (ARPA) are also focused on sediment and water quality and ecotoxicological assessment.

Such characterization plans are focused on the reconstruction of the contamination phenomena to obtain a baseline for feasible and sustainable decisions for site safety and/or remediation. Nevertheless, a comprehensive characterization should also account for potential morphodynamic variations induced by climate change drivers (e.g., extreme events as floods and storm surges, even enhanced by sea level rise) and anthropogenic modifications (e.g., realization of harbor facilities and infrastructures) as they can affect the environmental fate, transport, speciation, bioavailability, toxicity, and inventories of contaminants in estuarine and marine areas^7,8^ as well as modify the resilience of common sediment remediation technologies^9^.

Therefore, in this study, a set of guidelines is proposed to define an integrated geo-environmental characterization for marine and coastal contaminated sites, accounting for the definition of both current and expected morpho-dynamic settings. In detail, the proposed guidelines are focused on:

1. The definition of the geological sensu lato model
2. The definition of the anthropogenic impacts on marine and coastal sediments
3. The definition of the expected morphodynamic variations

Based on the assumption that the geological setting sensu lato (considering geological, geomorphological, and sedimentological aspects) plays a key role in the contaminants’ distribution and accumulation, tailored surveys are suggested to obtain both the site-specific geological model and the geometric relationships among different sedimentary units. The latter may be used to evaluate the potential spatial distribution of contaminants. To obtain a comprehensive assessment of the impact of anthropogenic activities, tailored surveys are also required to define morphological modifications on the seafloor. Finally, as the integrated characterization cannot disregard from the assessment of the expected variations in physical processes induced by climate change and anthropogenic interventions, the proposed guidelines suggest specific analysis and surveys for supporting the evaluation of local morphodynamic variations.

The outcomes of this study, which are also provided in the form of infographics, are expected to be a useful investigation tool to support the sustainable management of the marine and coastal areas strongly affected by anthropogenic impacts. The need for an integrated coastal characterization that takes into account the severe impacts of human activities, climate change, and shoreline dynamics on coastal economic development and growth, as well as on marine ecosystems, is highlighted by the European Directive 2014/89/EU (Maritime Spatial Planning), which requires Member States to contribute, through the implementation of tailored maritime spatial plans, in the preservation, protection, and improvement of the marine environment, including resilience to climate change drivers. Similarly, according to the European Union Marine Strategy Framework Directive (2008/56/CE), implemented in Italy with the Legislative degree n. 190/2010, Member States are called to act for ensuring a good ecological status of the marine water. To this aim, eleven descriptors have been proposed. They include the preservation of the sea floor integrity (Descriptor 6), the maintenance of favorable hydrographic conditions (Descriptor 7), the low concentration of chemical pollutants (Descriptor 8), and the limited quantities of marine and coastal litter (Descriptor 10). Therefore, a comprehensive characterization of contaminated coastal sites should provide key elements to comply with such descriptors.

**Figure 1:**
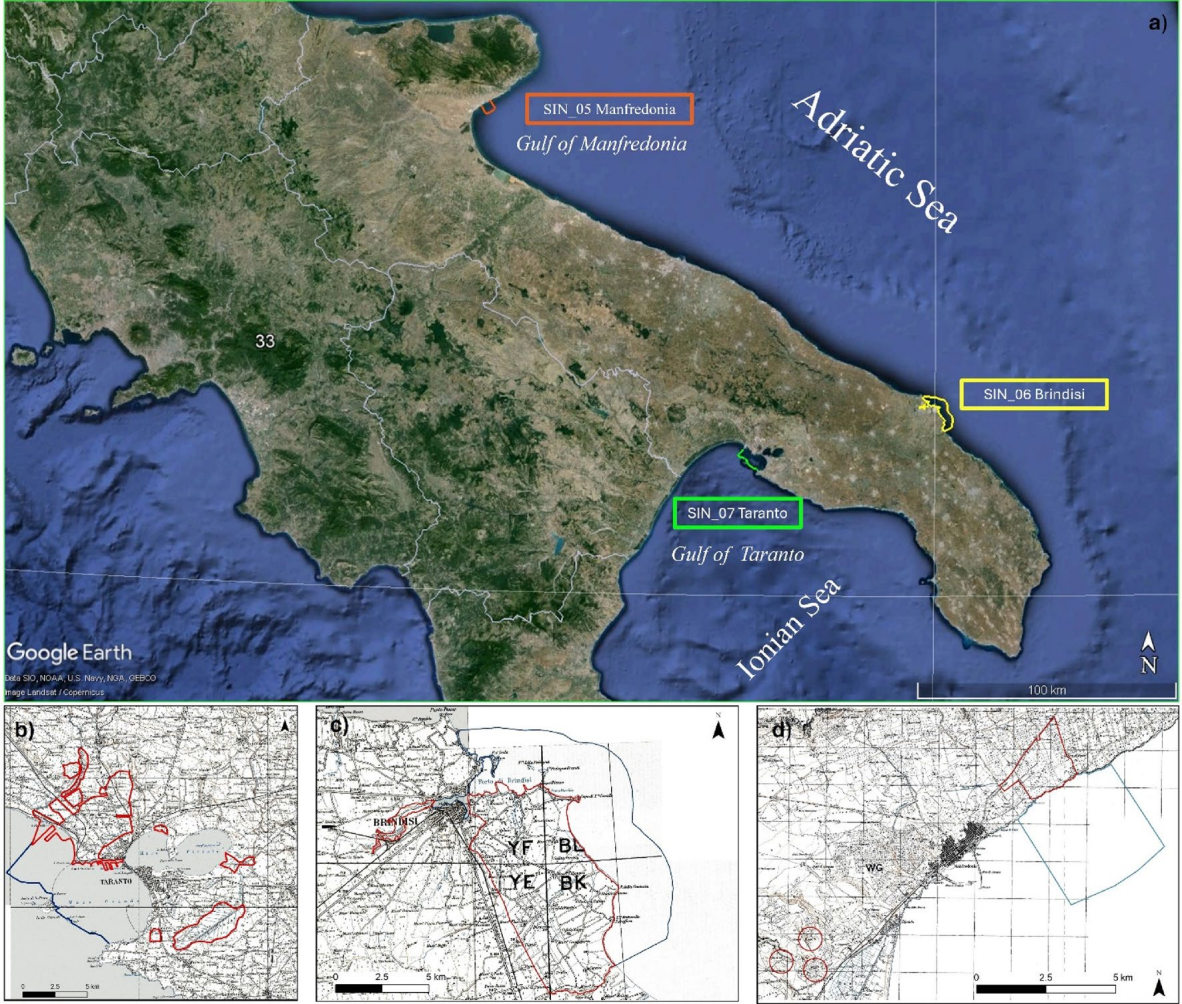
(**a**) Geographic location of the Apulian coastal SINs. SIN_05 “Manfredonia” and SIN_06 “Brindisi” are located along the Adriatic coast whereas SIN_07 “Taranto” overlooks the Gulf of Taranto. Background image was exported from Google Earth and modified by the authors. Perimeters are reported in (**b**) for SIN_05, (**c**) for SIN_06, and (**d**) for SIN_07. Blue lines identify the marine area included in the SINs perimeters whilst red lines identify the in-land areas. SINs perimeters in (**b**–**d**) were provided by the Italian Ministry of the Environment.

The research is based on the analysis of previous characterization plans proposed for the SINs located in the Apulia Region (SIN_05 “Manfredonia”, SIN_06 “Brindisi”, and SIN_07 “Taranto”–whose geographic location and perimeter are shown in Fig. 1). SIN _07 includes Taranto marine basins, called Mar Grande (“Big Sea”) and Mar Piccolo (“Little Sea”), which is divided by a promontory in two connected bays (the First Bay—“*Primo Seno*” and the Second Bay—“*Secondo Seno*”). Due to its peculiar hydrogeological and geomorphological characteristics, the Taranto coastal area has typical lagoonal features, which have favoured a high local biodiversity^10^. Nevertheless, due to the high urbanization and industrialization, the Taranto area is characterized by high contamination affecting all the environmental matrices^11–14^. SIN_06 occupies a land surface of 5700 ha, partly destined for agricultural use (vineyards and vegetable and cereal cultivation). Such areas receive pollutants produced by the chemical and industrial plants and by the Brindisi Harbour, one of the largest ports in the Mediterranean Sea, and related facilities^15,16^. SIN_05 is located in the Manfredonia Gulf (Adriatic Sea). The marine coastal area in the SIN perimeter has an extension of about 4km along the coast, for a total area of 860ha. The area includes industrial plants, which were devoted to the production of nitrogenous fertiliser and various chemical products, and landfills built in calcarenite quarries used in the 1970s as storage sites for unauthorised solid urban waste.

The main characteristics and activities already performed in the Apulian SINs are synthetized in^17–19^.

## Proposal of guidelines of investigation for the integrated geo-environmental characterization of highly contaminated coastal sites

The integrated analysis and characterization of the highly contaminated coastal sites will support the definition of tailored management measures and reclamation activities. From the geological s.l. point of view, these aspects include:

- The definition of the site-specific geological sensu lato model;
- The definition of anthropogenic impacts on marine and coastal sediments over the time;
- The definition of expected morphodynamic changes, also in relation to the impact of ongoing climate change on marine processes and their consequences on pollutants’ distribution.

**Figure 2:**
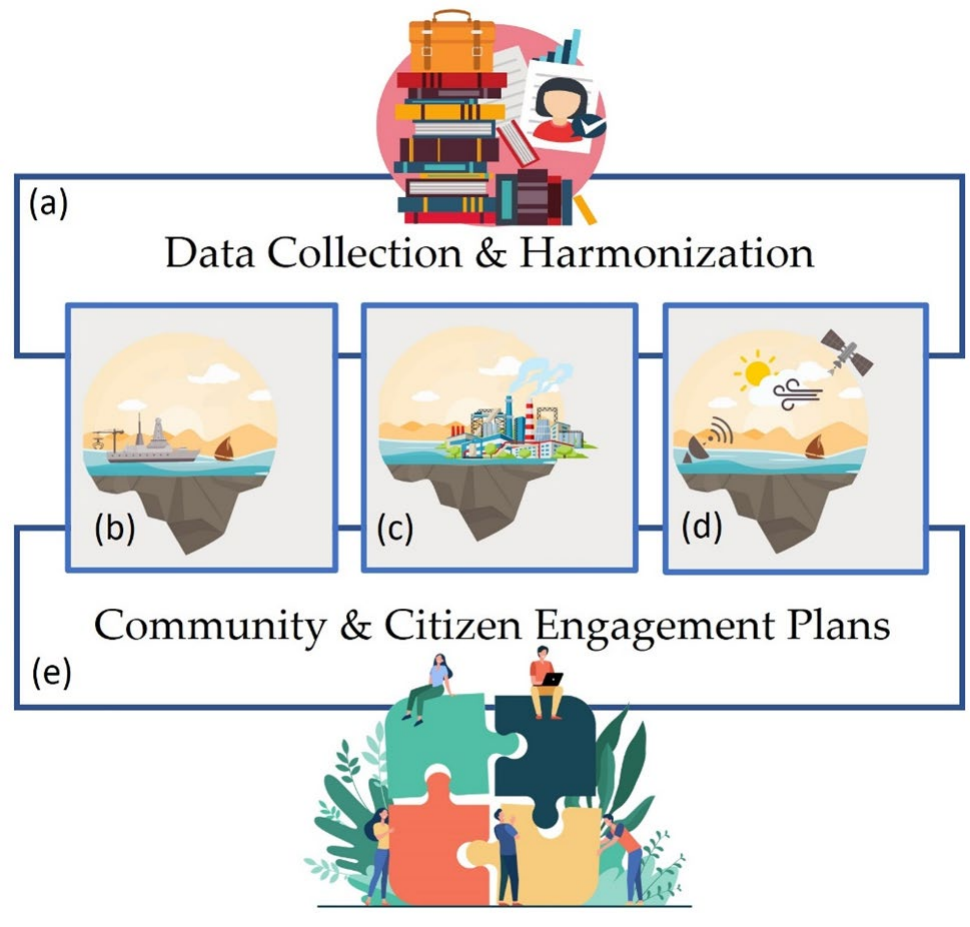
Schematization of the investigation phases proposed as guidelines to be applied for the characterization of highly contaminated coastal sites: (**a**) preliminary scoping phase, (**b**) definition of the geological sensu lato model, (**c**) definition of anthropogenic impacts on marine and coastal sediments, (**d**) definition of the expected morphodynamic variations, (**e**) dissemination phase. Clipart images in “(**a**)” and “(**e**)” were freely downloaded from freepik.com (authors: GraphiqaStock/Freepik, avectorjuice/Freepik). Images in “(**b**)”, “(**c**)”, and “(**d**)” were designed by Giovanni Pellegrino by using clipart images freely downloaded from canva.com.

Furthermore, the increase in citizen and policy-maker awareness is crucial to fostering capacity building and dissemination of scientific evidence on detected risks in contaminated sites. For this reason, the definition of community engagement plans represents the final step of the process. In Fig. 2, a synthesis of the investigation phases suggested for the achievement of the integrated characterization is shown.

As a preliminary scoping activity of each proposed phase, it is recommended to collect, reorganize, and harmonize information and data available from previous studies, surveys, and monitoring activities. To this end, scientific literature databases such as Google Scholar, ResearchGate, Scopus, and Web of Science are suggested to collect relevant research papers. Furthermore, grey literature, national and regional reports, and site-specific monitoring plans play a strategic role and should be also consulted. This preliminary activity is expected to lead to the design of a specific GIS geodatabase that will support the assessment and the recognition of available information as well as the spatial analysis of available data.

In the following subparagraphs, the objectives of each investigation phase are introduced.

### The definition of the geological sensu lato model

The geological sensu lato (s.l.) model, which includes geological, sedimentological, and geomorphological aspects, represents the basis for the identification of the different sedimentary units and their geometric relationships, as well as for the evaluation of the thickness of superficial deposits. Based on the assumption that the geo-morphological setting of each investigated area affects the physical processes and the distribution of the contamination in all the environmental matrices, the geological s.l. model is considered a key feature for supporting integrated geo-environmental analysis and defining expected variations. This investigation phase is aimed to identify in situ surveys and lab analysis to be considered as guidelines for the definition of the geological s.l. model with specific reference to marine investigations. Based on the desk review of national and regional monitoring plans, it emerged that in the case of semi-enclosed basins, the entire marine area is suggested to be analyzed, whilst, in the case of coastal sectors with a wider extent, only the area considered potentially affected by contamination, need to be analyzed.

### The definition of the anthropogenic impacts on marine and coastal sediments

This investigation phase proposes a set of activities aimed at defining the direct and indirect anthropogenic impacts on marine and coastal matrices. Direct impacts can be defined through geo-chemical analyses to estimate concentrations of organic and inorganic pollutants (including microplastics) in surficial and sub-surficial sediments. Indirect impacts, which include anthropogenic footprints on seabed morphology, i.e., the impact of maritime activities carried out in coastal areas (such as shipping, fishing, and shellfish farming) and the dumping of litter and mega-litter, can be assessed through the interpretation of high-resolution geophysical data (i.e., acoustic and magnetometric surveys). In this context, special attention is given to the geomorphological aspects, due to their influence on sediments and contaminant distribution and accumulation processes. Anthropogenic impacts affect both the sea bottom and the sub-surficial sediment layers. Therefore, the suggested guidelines are focused on the analysis of the 3D spatial distribution of the contamination. According to the requirements of the MSP Directive (Art. 5) and MSFD (Annex III), Member States shall consider environmental aspects to support sustainable development and growth in the maritime sector and for the determination of good environmental status.

### The definition of the expected morphodynamic variations

Based on the assumption that both anthropogenic activities along the coastal stretch and ongoing climate change are expected to induce cascading effects on physical coastal processes (e.g., current-sediment interactions, erosion processes, etc.) and their related spatial and temporal patterns, this investigation phase is aimed at defining surveys for the monitoring and analysis of the potential morphodynamic variations in marine variables.

Therefore, the suggested guidelines focus on data to be used for defining potential physical impacts of climate change and anthropogenic interventions on dynamic conditions in contaminated coastal sites in order to predict the influence of such modification on local erosion/accretion processes and contaminated sediment dispersion.

### The definition of community engagement plans

A participatory stakeholder process can lay the foundations for more extensive citizens’ participation in public processes resulting in the identification of collectively accepted site-specific solutions for the management of highly contaminated coastal sites. Stakeholder engagement in participatory decision-making at the local and regional scale is therefore considered fundamental to mitigate environmental and health risks and, for this reason, the most innovative methods to be implemented for the definition of tailored community engagement plans are proposed. This aspect meets the MSP Directive requirements, according to which Member States shall establish means of public participation by informing all interested parties and by consulting the relevant stakeholders and authorities (Art. 9) and shall organise the use and sharing of the best available data and information (Art. 10).

## Overview of suitable investigations

### Activities envisaged for the definition of the geological s.l. model

The scoping activity of this investigation phase is aimed at the acquisition of geological and geomorphological maps as well as at the recognition of data derived from previous characterization procedures (such as geophysical surveys, sediment sampling, and maps). This phase will set the formalization of existing site-specific knowledge and will support the identification of tailored surveys and analyses for data integration and knowledge gap filling.

**Figure 3:**
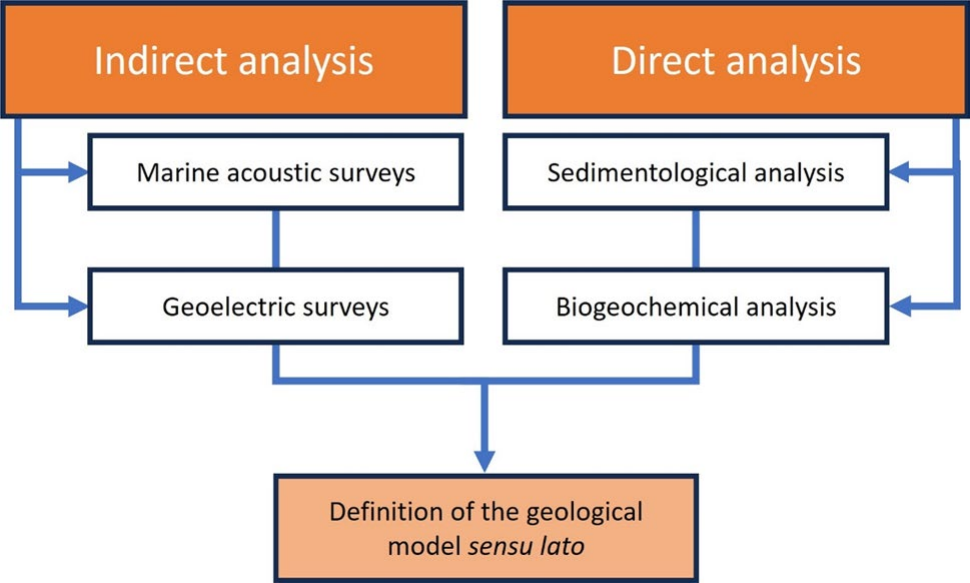
Synthesis of activities that are performed to define the geological s.l. model.

Based on a deep desk review of the surveys applied for the definition of the geological, sedimentological, and geomorphological setting of the coastal contaminated sites, the following categories of analysis (Fig. 3) are suggested for the definition of the geological s.l.model:

Indirect (geophysical) analyses, which include acoustic systems (seismic-stratigraphic and morpho-bathymetric surveys) and geoelectric surveys required to define over a wide coastal area the geological, stratigraphical, and geomorphological architecture;

Direct analyses, which include sedimentological, geochemical, mineralogical, paleontological, and biogeochemical analysis required to define the lithostratigraphic characteristics of rock outcrops and marine substrate as well as the inorganic characteristics of sediments.

Indirect analyses, which involve the use of acoustic transmission, receiving, and measuring instruments as well as high-precision geographic positioning systems on survey boats, have been widely performed for gathering surface and subsurface geological and geotechnical data in marine and coastal environments for several decades^20–22^. 

#### Marine acoustic surveys

**Figure 4:**
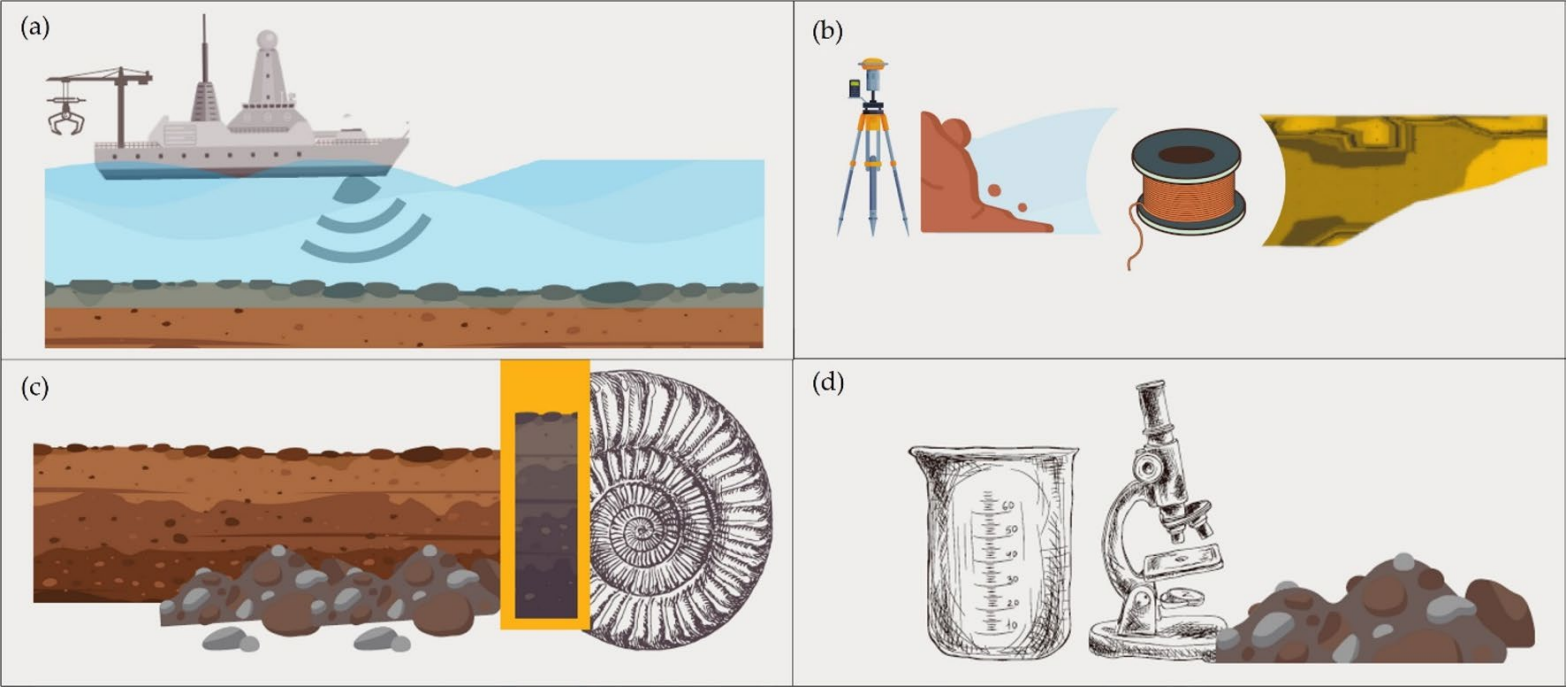
The activities envisaged for the definition of the geological model of coastal contaminated sites include (**a**) marine sediment sampling and morpho-acoustic surveys, (**b**) geoelectric surveys, (**c**) sedimentological analyses, and (**d**) geochemical, mineralogical, and biogeochemical analyses. The comprehensive proposal of activities and surveys suggested for the definition of the geological s.l. model is graphically shown in Appendix 1. Images were designed by Giovanni Pellegrino by using clipart images freely downloaded from canva.com.

Marine acoustic investigations are commonly used in marine surface and sub-surface exploration to recognize the morpho-bathymetric characteristics and the seismic-stratigraphic structure of geological units (Fig. 4a).

In detail, surface acoustic surveys (i.e., Single/Multi Beam Echosounder, Side Scan Sonar) allow obtaining the bathymetry and landform of the seabed while sub-surface acoustic surveys (i.e., Sparker and Sub Bottom Profile) allow defining the sub-bottom stratification as well as the engineering properties of the materials. Considering that the sound velocity in water is known, marine acoustic surveys are based on the evaluation of the elapsed time of an acoustic pulse from a generating transducer to the seafloor and back^23^. The accuracy of the detection is strongly correlated with the wavelength of the transmitted signal: the shorter the wavelength (high frequency), the higher the resolution, and vice versa. This means that high-frequency waves have a good resolution but a poor ability to penetrate the seabed, while low-frequency waves have a great ability to penetrate the seabed but a low resolution. Suitable marine acoustic surveys are briefly described in Table 1.

**Table 1:** Summary of the marine acoustic surveys suggested for the acquisition of data to be used for the definition of the geological s.l. model.

Survey	Objectives	Notes
Single beam echo sounder systems (SBES)	SBES allow to define water depth and bathymetric maps	Single-beam systems are preferred to survey very shallow water
Multibeam echo sounder systems (MBES)	MBES allow to obtain sea floor morphology and elevation	The use a fan of narrow acoustic beams allow also to infer the substrate^20,24^
Side Scan Sonar (SSS)	SSS survey the seafloor surface with very high resolution but with a low penetration^25^	It is possible to distinguish between high-frequency systems, which work at a dual frequency, between 100 and a maximum of approximately 1000 kHz, and low-frequency systems, which operate down to 30 kHz^22^
Mono-channel seismic reflection surveys: sub-bottom profiler (SBP)	SBP systems allow to obtain information about the strata and to detect submarine stratigraphic features buried within the stratum^26^	SBP signals are generally presented as images whose interpretation allows the identification and mapping of the main seismic horizons that, together with the first reflector representing the seabed, constitute the upper limits of seismo-facies
Mono-channel seismic reflection surveys: sparker (SPK)	SPK systems allow to define the stratigraphic structure beneath the seafloor due to the high-resolution sub-bott penetration	SPK profiles are obtained from intermittent seismic (impulsive) sources and typically use sources that operate at frequencies ranging between 50 Hz and 4 kHz^27^
Multi-channel reflection seismic surveys	Multi-channel surveys are used to generate sound that penetrates several kilometres beneath the seafloor	Multi-channel reflection seismic surveys use arrays of multiple hydrophones groups referred to as channels, positioned along a streamer ranging in length from ~ 100 m to more than 10 km long for modern multichannel seismic data acquisition^27^

#### Geoelectric surveys

Geoelectric prospecting (marine and terrestrial) is an indirect investigation aimed at defining the electrical resistivity of rocks and water bodies and, therefore, it allows the identification of the main lithological discontinuities and the fresh-saltwater interface (Fig. 4b). Measurements for resistivity surveys are made by the streaming current into the ground through two electrode types (potential electrode and current electrode) and measuring the resulting voltage difference. Different inter-electrode spacing is used to allow a multiscale reconstruction of the distribution of electrical properties in the subsurface. The geoelectric surveys can be performed based on a number of different configurations of the electrodes, each of them having its own calculation method to know the value of thickness and resistance of rock types beneath the surface^28^. Geoelectric surveys are used in a wide range of environmental issues in many complex geological and hydrogeological settings^29^. Furthermore, according to a recent literature review carried out by^28^, geoelectric surveys are a fairly effective and reliable geophysical tool to detect the presence of groundwater, lithology, and rock stratigraphy and can be carried out with simple equipment, in a short time and low cost.

#### Sedimentological analyses

Sedimentological analyses allow the definition of stratigraphic, radiographic, and radiometric characteristics of the marine and coastal sampled sediments and the correlation with seismic interpretations (Fig. 4c). Shallow and deep sediments may be sampled by using different survey tools which allow the collection of both surface (from the sediment–water interface) and sub-surface samples. The sampling methodological approaches and techniques, which include grab and core samplers, were reviewed in^30^. Due to the low penetration depth, samples collected by grabs are used to define the textural characteristics of the surface sediments; in this case, the sedimentation history is not recognizable. On the other hand, corers, which include gravity corers, piston corers, box corers, and vibrocorers, are the tools used for collecting subsurface and deep sediments. Core sampling allows for careful analysis of the sediment vertical profiles and, therefore, marine sediment cores represent the source of information to define seabed character as well as the depositional history and environmental change that occurred in the investigated site^31^. Cores characterization, mostly based on granulometric, magnetic, and radiographic analyses, coupled with the evaluation of the biological contents and their radiocarbon dating, supports the definition of the main stratigraphic units and their geometric assets, allowing their correlation with different sources of data (e.g., seismic data). The grain-size analysis of marine sediment samples, both surficial and sub-surficial, is carried out by following standard procedures for both grain fractions with diameters higher than 63 µm and < 63 µm, being the limit between coarse fraction (mainly constituted by sand and gravel) and the fine fraction (mainly constituted by silt and clay). Once sieved, each held of the coarse fraction is weighted and the results are statistically analyzed in order to evaluate the main textural parameters: mean size, sorting, skewness, and Kurtosis^32^.

#### Geochemical, mineralogical, and biogeochemical analyses

Laboratory analyses, which include geochemical, mineralogical, and biogeochemical analyses, are performed to characterize sediments from an inorganic component perspective (Fig. 4d). In addition, sample dating (Radiocarbon dating-14C) and bio-geochemical analyses (e.g., palynological analysis) support the reconstruction of the paleo-environmental conditions that characterized the investigated site and therefore allow the definition of the sedimentary environment. The analyses that can be envisaged on marine sediment samples include those reported in Table 2.

**Table 2:** Summary of the analyses suggested for the sediment samples characterization.

Analysis	Objectives	Notes
X-ray fluorescence	XRF allows non-destructive, nearly continuous, and relatively fast analysis of the elements and trace metals in sediment samples	Sediment's chemical composition is directly analized on the sample surface of a split sediment core. The impact of physical properties on XRF core scanner was investigated by^33^
X-ray powder diffraction	XRPD allows to describe the characteristics of crystalline structure and determine the mineralogy of finer-grained sediments, especially clay minerals^34^	The description of the clay content supports the analysis of the paleo-climate and paleo-environmental conditions that characterized the investigated site^35^
Transmission electron microscopy and scanning electron microscopy	TEM and SEM allow the chemical, morphological, and diffractometry analysis of the sediment samples	A deep description of the SEM and TEM common applications is applications given in^36^
Macrobenthic component	The characterization of fossil and subfossil macrobenthos, allows to define past environmental conditions	The characterization of the fossil malacofauna coupled with radiometric dating (14C) allows to estimate the average rate of local sedimentation
Marine palynological analyses	MPA are used to reconstruct the type of sedimentary environment	Allow to estimate the relative importance of fluvial and pelagic inputs to a sedimentary basin^37^

### Activities envisaged for the definition of the anthropogenic impact on marine and coastal sediments

The analysis of the spatial distribution of the contamination in marine and coastal sediments starts with the definition of the site-specific background values that characterize the investigated coastal site as well we the knowledge of the historical anthropogenic activities carried out in the selected coastal sites. Therefore, the scoping phase is aimed at collecting the results of previous characterization activities carried out to define both the site-specific background values and the contamination level.

#### Chemical pollution

The chemical characterization of surficial sediment allows to determine the direct impact of anthropogenic activities in terms of pollutant concentrations (organic and inorganic pollutants). The analysis of the chemical contamination of marine sediments, as well as the extent of chemical pollution in sediments and its potential sources, is generally supported by the application of specific environmental indicators that allow the evaluation of the potential impacts of specific contaminants on the biological components. Indicators include the geo-accumulation index (Igeo^38,39^), the enrichment factor (EF^40^), the contamination factor (Cf^41^), the modified contamination degree (mCd^42^), and the pollution index (PLI^43^). As highlighted by^44,45^, the application of the pollution indices requires the calculation of contaminant concentrations and the identification of background values. The latest refers to the concentration of metals in pristine sediment, unaltered by anthropogenic activities. They can be calculated by both empirical and statistical methods.

**Figure 5:**
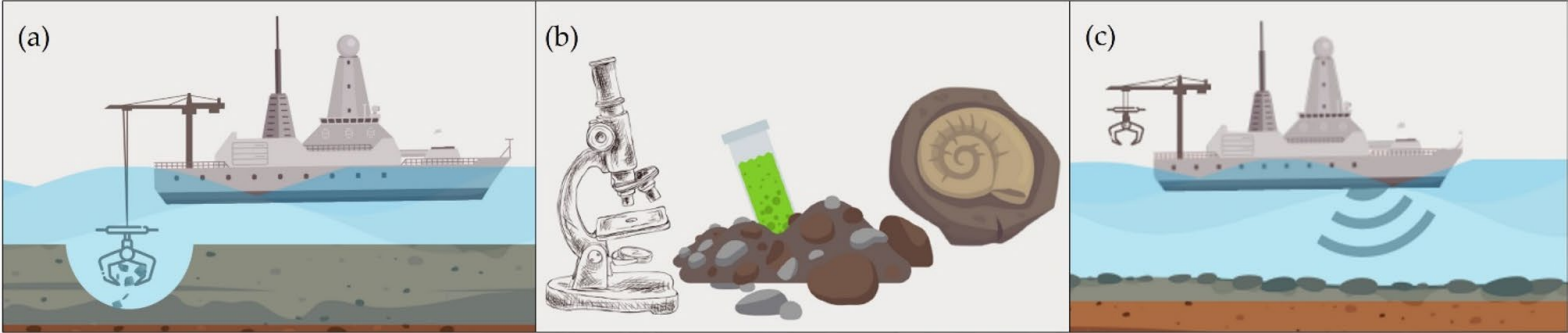
Activities envisaged for the definition of anthropogenic impact on marine and coastal sediments include (**a**) sediment sampling, (**b**) geochemical analyses, and (**c**) geophysical surveys. The comprehensive proposal of activities is graphically shown in Appendix 2. Images were designed by Giovanni Pellegrino by using clipart images freely downloaded from canva.com.

To this aim, the identification of suitable sampling sites is a fundamental requirement and it can be supported by the interpretation of marine geophysical data (Fig. 5a). Suitable sites for sediment sampling are defined according to the surficial seabed sediment properties. To this aim, the exploitation of SSS surveys can be very useful since backscatter data allow to map surficial seabed sediment properties relevant to sediment dynamics, including sediment grain size and seabed roughness^46^.

Once collected, sediment samples are stored following standard techniques and prepared for further chemical and toxicological analyses (e.g., U.S. EPA, 2001; Italian Ministerial Decree 7/11/2008). The chemical characterization of surficial sediment allows to determine the direct impact of anthropogenic activities in terms of pollutant concentrations. On the other hand, the analysis of the pollutants distribution along the vertical sediment profile allows to assess the occurrence of anthropogenic reworking processes and the site-specific background values^47,48^.

Samples preparation and chemical analyses are performed following national directives (e.g., Italian Legislative Decree, n. 152/06) and international procedures (e.g., EPA methods). Concentrations are determined by analytical methods (Fig. 5b), which include techniques of atomic spectrophotometry (AAS, GF-AAS) and plasma (ICP-MS, ICP-AES). A critical review of current and emerging capabilities of analytical methods used for marine sediment analysis was proposed by^49^ while the assessment of chemical-based sediment quality methodologies was published by^45^.

In the Italian legislation, a quality approach for the assessment of the potential impacts of pollution on human health and the environment has been established with Law N. 172/2015 (Implementation of 2013/39/EU). Furthermore, in the Italian Legislative Decree N. 152/2006, national limits for contaminant concentrations are provided for both sites devoted to commercial and residential use and industrial sites. In 2004, ICRAM evaluated site-specific action values for the analysis of marine sediments in several highly contaminated national sites. At the international level, sediment quality guidelines (SQGs) have been developed to deal with environmental issues and evaluate the adverse effects caused by contaminated sediments on biota^44^.

#### Microlitter pollution

The analysis of micro-litter (i.e., microplastics-MPs) in marine sediments represents quite a recent challenge^50^. The methodological procedure for the identification and classification of MPs in marine sediments requires preliminary sample preparation and pretreatment phases, which include the sieving, digestion, separation, and isolation phases^50^. Then, as a further step, the MP items are classified according to the polymer type. To this aim, different methods can be used (e.g., Raman, μRaman, FTIR microscopy, SEM)^51–53^. Innovative approaches are based on HyperSpectral Imaging (HSI) technique, which uses hyperspectral images acquired in the short-wave infrared range (SWIR: 1000–2500 nm) to characterize MPs in terms of abundance, distribution, category, morphological and morphometrical features, and polymer type^54–56^ In^57^ a review of 80 papers on microplastic particles in marine sediments for different sedimentary environments is proposed.

#### Human footprint on the sea-floor

Marine sediments are also indirectly impacted by anthropogenic activities, mainly related to maritime, shipping, and fishing activities as well as by the accidental and/or illegal release of waste and mega-litter, which cause remarkable morphological changes on the sea-floor^5,58–62^. The mapping process of such kind of anthropogenic footprints is supported by the marine morpho-acoustic surveys (SSS and MBES–Fig. 5c), whose signal interpretation allows the sea-floor characterization in terms of both abiotic and biotic properties (e.g., substrate types, benthic faunal elements) and anthropogenic traces and items identification^63,64^. In very shallow water conditions, marine drones can be also envisaged for data acquisition in areas where traditional boats are poorly maneuverable (0–20 m of depth). Such kind of drones are designed to be equipped with different morpho-bathymetric sensors^65,66^, allowing the object detection process^67^.

### Activities envisaged for the definition of expected morphodynamic variations

The alteration of energy and mass balances generated by anthropogenic interventions (e.g., the construction of coastal structures such as breakwaters, jetties, seawalls, and bulkheads) as well as by the influence of climate change on marine and atmospheric variables may cause variations in sediment transport and trigger contaminant mobilization mechanisms^68,69^. The activities envisaged in this investigation phase require as the scoping phase the collection of bathymetric and topographic data already available at the national, regional, and local scales as well as the acquisition of historical marine and weather data (i.e., waves, sea level, wind). This is because expected climate- and anthropogenic-induced variations include morphological changes that affect both the submerged and the emerged portion of a coastal area and it is therefore pivotal to have a baseline to which to refer. To this aim, national and regional repositories and online data platforms have to be consulted.

**Figure 6:**
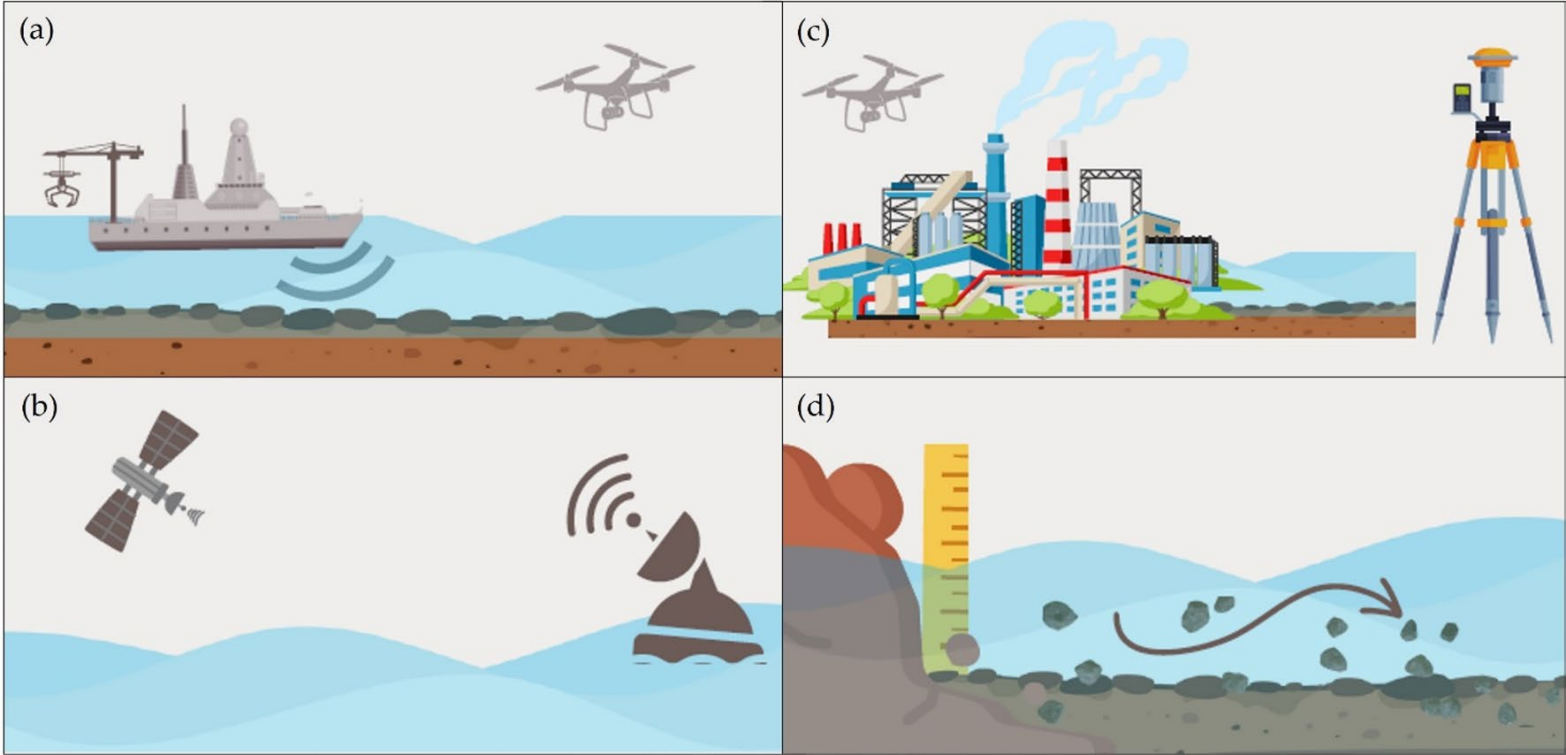
Activities envisaged for the definition of expected morphodynamic variations include (**a**) performing high-resolution topographic and bathymetric surveys, (**b**) modelling of future variations in marine parameters, (**c**) evaluation of medium- and short-term geomorphological modifications, and (**d**) analysis of expected variations in marine parameters. The comprehensive proposal of activities and surveys suggested for Stage 2 is graphically shown in Appendix 3. Images were designed by Giovanni Pellegrino by using clipart images freely downloaded from canva.com.

From the operative point of view, high-resolution topographical and bathymetric data have to be collected (Fig. 6a). Topographic data in coastal environments are usually obtained by means of direct surveys, terrestrial laser scanning, and aerial surveys^70^. Direct surveys (e.g., GNSS) are time-consuming and only allow surveys of limited coastal sectors. On the other hand, TLS surveys, generally performed by phase difference laser scanners, allow for covering wide coastal sectors but then require high post-processing time^71^. Recent advances in the availability of Unmanned Aerial Systems (UAS) and drones with high performance in terms of flight duration, sensor availability, and mapping resolution provide a great opportunity for obtaining in a relatively short time Digital Surface Models, allowing their comparison over time. Compared to other airborne photogrammetry surveys, the error associated with UAV photogrammetry is smaller and, furthermore, the area covered by drones is larger than the one that can be investigated by terrestrial laser scanning^72^.

For what concerns the bathymetric surveys, single and multi-beam acquisitions are suggested (cf. section “Activities envisaged for the definition of the anthropogenic impact on marine and coastal sediments.”). Also in this case, marine drones can be envisaged to survey very shallow water.

High-resolution coastal topographic and bathymetric models can be exploited for the analysis of the evolution of coastal morphologies (e.g., dune elevation, evolution of submerged bars^73^) as well as for the evaluation of ongoing processes (e.g., local subsidence trends^74,75^) and for the assessment of the potential impacts of coastal hazards (e.g., storm surges, sea level rise^76–78^).

Among the proposed activities, the analysis of past and recent morphological variation, in terms of erosion and accretion trends, plays a remarkable role in supporting the evaluation of predominant coastal processes (Fig. 6b). In detail, shoreline position over time (i.e., long-term: > 30 years; medium-term between 30 and 10 years; short-time: < 10 years) is evaluated by using different topographic sources (aerial-photos, ortho-photos, Google Earth images, ESRI images), by direct in-situ surveys (DGPS and RTK), and, more recently, by the automatic extraction of the shoreline from satellite data^79^. Different coastal forms (high tide water level, low tide water level, wet/dry line, seaward stable dune vegetation line) can be used as shoreline proxies to identify the shoreline position^80^. Then, shoreline positions are compared by GIS-based tools (e.g., Digital Shoreline Analysis System^81,82^) and results can be expressed in terms of both distance (e.g., Net Shoreline Change, which expresses the total movement between the two shoreline positions) and rates (e.g., Linear Regression Rates, which is expressed by the slope of the linear regression evaluated through all the available shoreline positions). Based on the results of such analysis, erosion/accretion processes along the investigated area are determined.

The analysis of expected morphodynamic variations cannot be disregarded from the analysis of the ongoing and future trends of marine and weather parameters that, directly and indirectly, influence the coastal systems' short- and long-term evolution (Fig. 6c). For what concerns the ongoing trends, national repositories can be consulted to collect data on parameters generally collected by national environmental agencies for scientific and monitoring purposes (e.g., wave, current, sea level, wind). Furthermore, the installation of in situ instruments will allow continuous monitoring^83^.

As far as the expected variations in marine and weather parameters are concerned, the comparison between historical data (baseline period) with future projections obtained from very high-resolution models, which are available at the global and regional scale, has to be performed^84,85^. Therefore, expected future variations are generally expressed in terms of anomalies with respect to the baseline period.

Both current and future trends in marine and weather parameters are exploited to model and assess the potential consequence that may be induced on the coastal areas (Fig. 6d), by applying high-performing hydrodynamics models, such as DELFT3D, MIKE21, SWAN, and tools therein^86^.

A remarkable issue that is expected to affect in the short, medium, and long term the contaminated sites located along the coastal zone is represented by the rising in sea level, which is considered  as one of the main consequences of increasing global temperature. In fact, the expected sea level rise may induce morphodynamic changes, resulting in loss of exploitable land and submersion of low-lying coastal sectors^87^. Many studies have been focusing on the definition of tailored methodologies for the identification of areas prone to be affected by RSLR. Such kind of analysis allows mapping areas topographically lower than expected sea levels. To this aim, global sea level future projections are downscaled at the local scale in order to account also for vertical ground movements which, in subsiding areas enhance the relative sea level rise. To this aim, satellite data have recently been exploited to assess local VGMs^75^.

One of the most applied procedures for the identification of susceptible coastal areas is represented by the bathtub method, a GIS-based approach that considers all the areas below a user-specified elevation as being flooded. The only data required as input for this method is a high-resolution and highly accurate Digital Elevation Model (DEM). Nevertheless, such a static method may overestimate the results^88^. To cope with such limitations, high-performing numerical models are suggested to investigate with higher accuracy the sea level rise impacts. Nevertheless, numerical models require high-performing tools and are time-consuming therefore, the extent of the area that can be analyzed is lower.

## State of the art of activities performed in the Apulian coastal sites

For what concerns the Apulian coastal SINs, it is worth highlighting that high-resolution surveys and sampling have been recently carried out for the definition of the geological model of the sea basins of the Taranto area (Table 3). Such kinds of studies have led to the identification of the main lithostratigraphic units characterizing the Mar Piccolo and Mar Grande basins. A detailed description of the main outcomes derived from the interpretation of the most recent data is reported by^18,78,89,90^. One of the major aspects highlighted in these studies is related to the definition of the local morpho-evolutive trends as well as the main environmental modifications that occurred since the Last Glacial Maximum. Furthermore, the analysis of both core and surficial sediment samples from the Mar Piccolo basins allowed a descriptive atlas of pollen useful to support the reconstructions of both local flora and vegetation and environmental and climate changes that occurred during the Holocene^91^.

**Table 3:** Summary of the surveys and analysis performed in the Apulian coastal sites (based on literature data and reports). Information in the three columns for each site indicates respectively the use of data for defining the geological model (GEO), the anthropogenic impact (ANT), and the expected morphodynamic variations (VAR). “n.a.” means that the survey is not available/not performed in the investigated sites (or it has not been used for the accounted analysis) while “hyphen” indicates that the survey is not useful data for the accounted analysis (GEO, ANT, VAR).

Surveys and analysis	SIN_05 MANFREDONIA			SIN_06 BRINDISI			SIN_07 TARANTO		
	GEO	ANT	VAR	GEO	ANT	VAR	GEO	ANT	VAR
Multi/single Beam	n.a	n.a	n.a	✔	n.a	n.a	✔	✔	✔
SSS	n.a	n.a	-	✔	n.a	-	✔	✔	-
SBP	n.a	n.a	-	✔	n.a	-	✔	✔	-
SPK	n.a	-	-	n.a	-	-	✔	-	-
Geoelectric	n.a	n.a	n.a	n.a	n.a	n.a	✔	✔	n.a
Magnetometric	n.a	n.a	-	n.a	n.a	-	✔	✔	-
Sediment sampling (≤ 3 m)	✔	✔	-	✔	✔	-	✔	✔	-
Sediment sampling (≥ 3 m)	n.a	n.a	-	n.a	n.a	-	✔	✔	-
Grain size	✔	-	n.a	✔	-	n.a	✔	-	✔
Chemical compound	✔	✔	-	✔	✔	-	✔	✔	-

Ongoing activities are being carried out in the framework of the national program for the elaboration of the geological map of Italy at the scale of 1:50,000, funded by the Italian ministry in 2019 (CARG Project). In detail, the SIN_07 is included in the area of “Foglio n. 493—Taranto” (Sheet 493). Activities scheduled in the collaboration agreement signed among the Italian Geological Survey, the Apulia Region, and the University of Bari included the surveying and data computerization of the whole emerged and submerged areas included in the reference area. The final version of the cartographic product will be released in 2024 and will be available on request at https://www.isprambiente.gov.it/Media/carg/puglia.html.

Similarly, in 2022, a collaboration agreement has been signed for the elaboration of the geological map of “Foglio n. 397–Manfredonia”, in which the SIN_05 is included. Also in this case, high-resolution indirect surveys as well as in situ terrestrial surveys will be performed to cover both the emerged and submerged territory. To date, the most updated geological data available for the area of SIN_05 refer to the studies and investigations carried out for the definition of the geological map at the scale 1:100,000 (Sheet 164 Foggia), in which only the bathymetric information is provided for the marine area.

SIN_06 “Brindisi” refers to the geological map of Sheet 204 (Lecce) and, partially, Sheet 203 (Brindisi). Updated geomorphological data relative to the marine and coastal area included in the SIN perimeter have been acquired during the survey carried out by ICRAM in the period 2008–2011. In this case, geophysical surveys (MBES, SSS, and SBP) were performed to define the bathymetry, morphology, and thickness of the incoherent sediment layer. Data interpretation has allowed to identify the presence of seagrasses, which were recognized both in the SSS and on the SBP data, and rocky outcrops, which were identifiable from the 3D analysis of the bathymetric data. Furthermore, the identification of the outcropping calcarenitic substrate allowed to define the sites where no sediment cores can be extracted and, therefore, to properly plan the sampling activities.

For what concerns the Apulian coastal SIN, a great effort has been posed for the SIN_07 “Taranto”, for which detailed characterizations have been performed to define both the direct (presence of inorganic and organic pollutants) and the indirect (presence of traces and objects) impacts on marine sediments and seafloor.

For what concern the analysis of chemical pollution, preliminary data were obtained through the surveys conducted by ICRAM in the period 2009–2010, during which both basins (“Mar Grande” and “Mar Piccolo”) were investigated. In 2013, the Regional Agency for Environmental Protection (ARPA) carried out further investigations covering the Mar Piccolo basin. In addition, several activities focused on the analysis of the spatial and vertical distribution of inorganic and organic chemical pollutants in different sediment layers were carried out for scientific purposes^13,14,47,92–96^. Ongoing research activities are also based on the identification of microplastic content in sediment samples collected both in Mar Grande and in Mar Piccolo basins.

Studies have focused on the identification of morphological anomalies on the seafloor in order to exploit the marine morpho-acoustic data for the identification of indirect anthropogenic impacts. Such kind of analysis has led to the definition of density maps of the Mar Piccolo and Mar Grande seafloor, by interpolating in the GIS environment all anthropogenic traces mapped from SSS and MBES data^58,60^. Similarly, in^71^, by analyzing MBES and SSS data, sea-floor features in the Primo Seno of the Mar Piccolo basin were detected, including both natural (i.e., karst springs) and anthropogenic features. A similar integrated geophysical study is presented in^65^, in which seismic-stratigraphic (SBP), morphologic (SSS), and magnetometric (MAG) surveys are used to identify two shipwrecks sunk in the Tyrrhenian Sea and to characterize both the lithology and morphology of the seafloor in the study area.

Such kind of studies highlight how the integration of multidisciplinary approaches (e.g., marine geophysical surveys, GIS tools, mapping procedures) is pivotal for the assessment of environmental criticalities and for the identification of hot-spot areas, i.e., areas with a higher density of anthropogenic objects, for which tailored management and remediation actions are a priority.

For what concerns the SIN_05 “Manfredonia”, chemical investigations performed on marine sediment samples by ISPRA in 2009, highlighted extremely high concentrations of mercury in some hot spots, both in the surface and sub-surface sediments. Furthermore, quite significant concentrations of synthetic organic compounds that can be traced to anthropogenic activities conducted in the area, such as caprolactam and polychlorinated biphenyls, have been also detected (approved by the “*Conferenza dei Servizi*” on 27/02/2009).

For the chemical characterization of SIN_06 “Brindisi”, ISPRA set up a monitoring program and defined site-specific action values to support the analysis of the contamination level (approved by the “*Conferenza dei Servizi*” on 22/04/2004). In this case, the marine area included in the SIN was divided into two main sectors: the harbor area and the coastal area. Due to the general low hydrodynamic conditions, the harbor area resulted to be more affected by chemical pollution, being the concentrations of several pollutants (Mercury, Lead, Copper, Zinc, Organochlorine—OC, pesticides, polycyclic aromatic hydrocarbon—PAH) above the site-specific action values defined by ISPRA.

As far as the analysis of the indirect impacts on the seafloor, there are no studies in the literature focusing on the identification of anthropogenic footprint and litter items in the coastal areas included in SIN_05 and SIN_06 using SSS, MBES, and MAG surveys. Nevertheless, recent studies focused on the analysis of litter items distribution along the coastal sector north to SIN_06^97–99^. In this case, the presence/density of anthropogenic waste on sandy beaches is used as a parameter to evaluate the environmental quality of the investigated coastal sites. Such an approach, which is based on the application of environmental indices and innovative procedures of analysis could be also exploited for the environmental quality assessment of the submerged areas.

Finally, for what concerns the analysis of expected morphodynamic variations, the coastal areas prospicient to the regional SIN_05 and SIN_06 have been analyzed in terms of the impact of future relative sea level rise. To this end, a well-consolidated methodological procedure applied in different Mediterranean coastal sites^100–106^, coupled with an innovative approach proposed for the evaluation of shoreline erosion, has been applied to identify zones prone to be inundated by future RSL and storm surge^107,108^. In Table 3, surveys and analyses performed in the Apulian coastal sites are summarized.

## Discussion

The activities proposed for the definition of the site-specific geological model are aimed at collecting geological, sedimentological, geomorphological, and geochemical data through both direct and indirect surveys.

It is interesting to note how the correlation between the two types of investigations (direct and indirect) allows to exploit the obtained data in a favourable way for the surveys' activities optimization.

**Figure 7:**
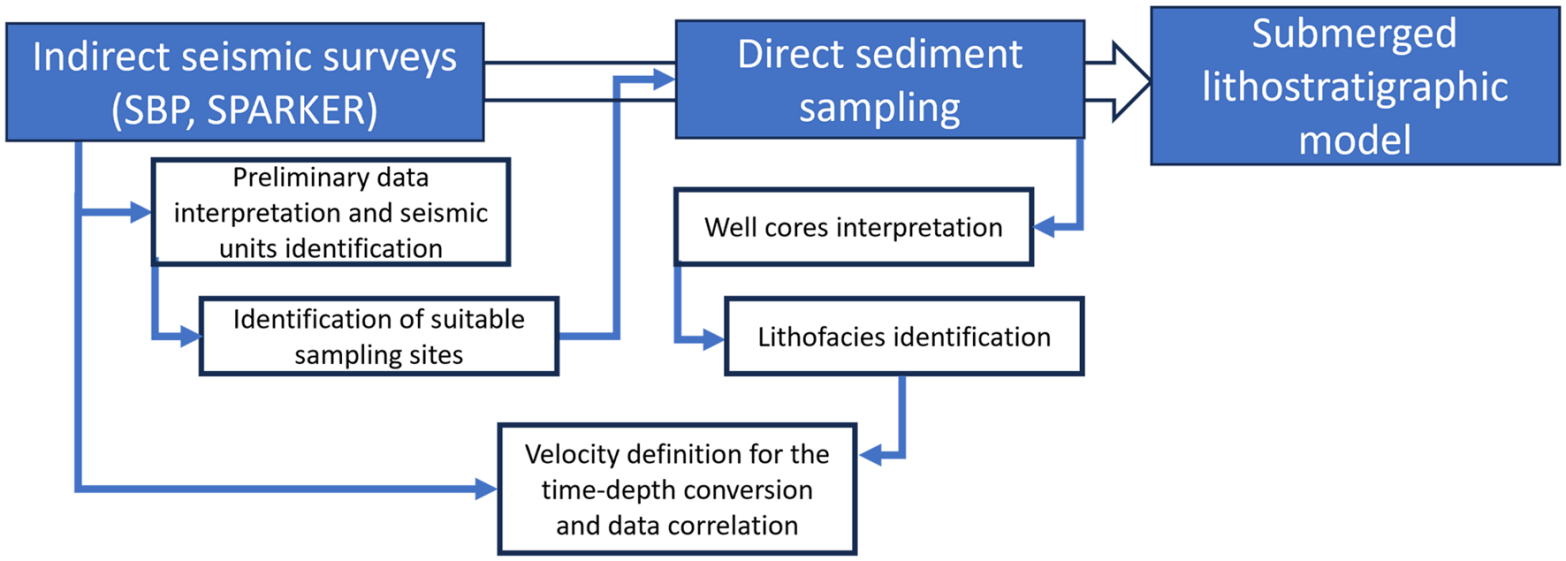
In the flowchart, the links between indirect and direct surveys proposed for the definition of the geological model are highlighted.

In fact, the indirect surveys, which include marine acoustic surveys (SBEC, MBEC, SSS, SBP, and SPK), are essential to investigate the sea-bottom morphology as well as the overall sub-bottom structure allowing the seafloor geological and geomorphological characterization and the definition of the structures and geometries within the stratigraphic succession. These aspects provide fundamental information for the accurate identification of sites where sediments could be sampled and for the definition of the most appropriate sampling mode. Therefore, the preliminary interpretation of marine acoustic data supports the feasibility of the in-situ sampling activities (Fig. 7). On the other hand, it is worth noting that seismic-stratigraphic data, which are recorded in the time domain, allow to obtain the acoustic impedance model but they do not allow the definition of specific lithologies^109^. Nevertheless, the macroscopic changes observable from the analysis of sediment cores (i.e., changes in texture, colour, porosity, water content, and grain size), expressed as well-logs, are in-depth domain and therefore allow to delimitate the sedimentary facies and correlate them to specific seismic units. Direct analyses performed on sediment samples allow to define the specific sound velocity and to convert the time data of seismic profiles into depth values and, therefore, to attribute a specific lithological and stratigraphic significance to each identified seismic unit (Fig. 7). Similarly, the interpretation of SSS imagery allow to identify seafloor setting such as muddy bottom, relief, rocky outcrops, and biological information (e.g., presence of phanerogam biocenosis^21^).

**Figure 8:**
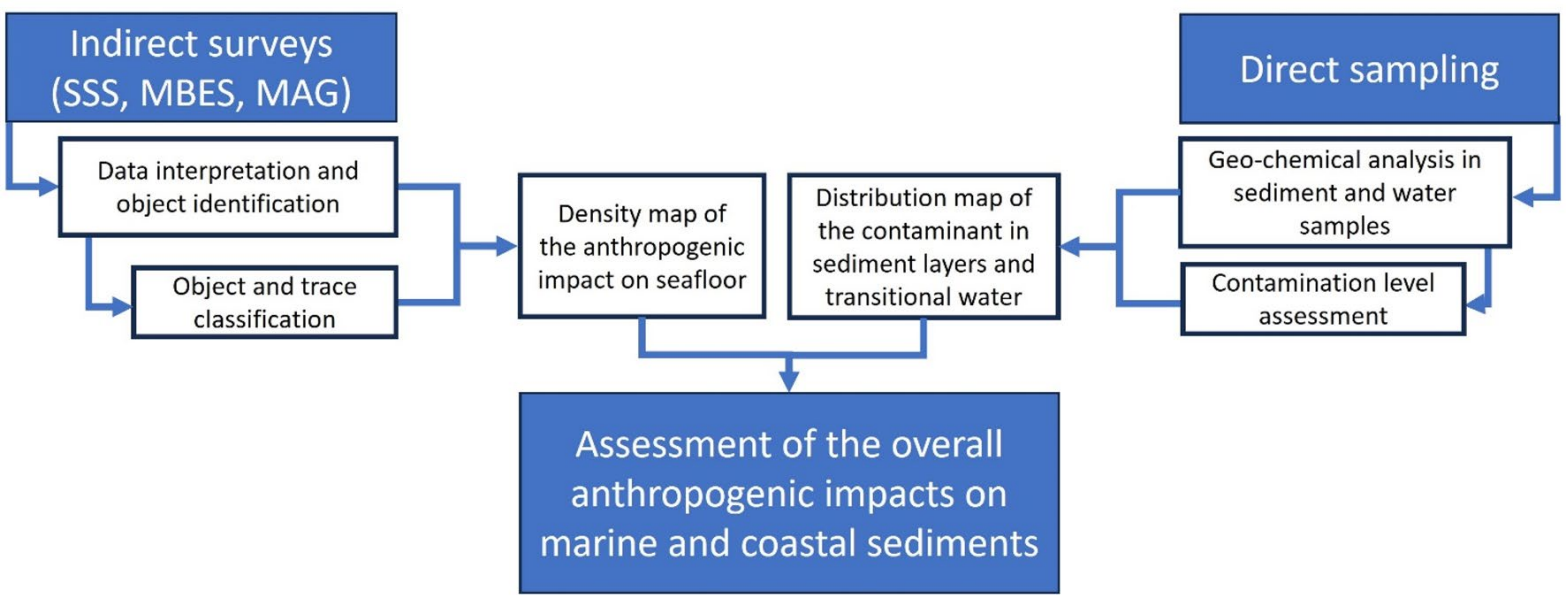
In the flowchart, the links between indirect and direct surveys proposed for the definition of the anthropogenic impact on marine and coastal sediments are highlighted.

The definition of the activities and procedures for the analysis of the anthropogenic impact on marine and coastal sediments highlights how data and samples collected to study and analyze the geological and sedimentological setting of a site can be exploited for the analysis of direct and indirect impacts on marine sediments (Fig. 8).

Indeed, the availability of deep marine sediment profiles allows to obtain detailed information on the sediment typology as well as pollutant and anthropogenic items (i.e. microplastics) therein^110^. This aspect is very relevant because it can support the definition of recent sedimentation rates and the identification of past mixing phenomena, as highlighted in^47^.

The potential 3D extent of the contamination can be reconstructed by the interpretation of SBP data, from which the seismic facies limits and the internal reflectors can be recognized allowing the correlation between specific lithology and corresponding lithofacies^89^. Similarly, the exploitation of morpho-acoustic datasets (SSS and MBES data) for the identification and mapping of traces and large litter items on the seafloor highlights the remarkable usefulness of marine surveys for the integrated characterization of marine environments^5,60,111^. This latter aspect allows to state that the efforts required for performing high-resolution marine morpho-acoustic data can be exploited for a dual purpose and therefore, such kind of data have a high suitability in supporting the geo-environmental characterization of coastal sites^17^.

Taking into consideration the evaluation of expected morphodynamical variations as a consequence of both natural and anthropogenic factors, one of the main aspects to be accounted for is the potential morphodynamical response that the coastal system could exhibit, which can act at both short and long-term. Coastal short-term response is expressed in terms of coastal erosion, which can be enhanced by marine-related processes (e.g., more intense storm surges) or by the construction of man-made structures (e.g., jetties, piers, and marinas). On the other hand, long-term coastal variations are mainly related to climate-related processes (e.g., sea level rise and variations in atmospheric parameters). In the case of sea-level rise, the expected coastal modification is represented by the submersion of coastal sectors whose elevation will be lower than the future sea level. Climate-related impacts also include variations in the marine parameters such as currents that, consequently, may influence the sediment transport and, therefore, the contaminants’ mobilization. As highlighted, such kind of analyses are oriented to support administrators in complying with the requirements of the European Directives aimed at ensuring clean, healthy, and productive seas and sustainable use of marine environments for current and future generations (MSFD) and at the climate risk reduction and adaptation. As emphasised in^112^, the sequence of “descriptor-criteria-indicatortarget-monitoring-measures-management” followed by the MSFD to address anthropogenic stressors cannot be separated form changes due to climate change. The proposed procedure therefore represents a step towards the evaluation of how climate change is directly linked to the Good Environmental Status Descriptors and to the definition of the impacts of coastal hydrodynamic changes due to climate variability and sea level rise on the environmental status of the coastal areas.

The proposed guidelines are oriented to the characterization of highly contaminated coastal sites (SINs) but the surveys can be also suitable to integrate the activities generally performed for the reconstruction of the contamination phenomena in potentially degraded sites. At the national level, a reference document for the environmental investigations in contaminated sites was published by the Italian Environmental Protection and Technical Services Agency in 2006. Nevertheless, no indications for the coastal and marine surveys were reported.

A preliminary analysis of the suitability level of the marine surveys and analyses was performed in^17^. The analysis has led to the identification of a number of surveys highly suitable for supporting both geological and environmental analysis.

Focusing on the operative application of the guidelines, a weak point could be represented by the availability of detailed data. Nevertheless, the recent development of national and international platforms dedicated to data sharing has made big data freely available for users. By way of example, the Copernicus services provide a huge set of data from the Copernicus space infrastructure. Copernicus C3S is dedicated to the climate-related dataset and provides data series and indicators for both the past and the future climate projections. The NASA web platform has been developed to allow the download of local sea level projections up to 2100, based on the climate scenarios adopted by the Intergovernmental Panel on Climate Change. Another relevant reference for imagery collection is represented by the Google Earth platform, whose data can be also imported in a GIS environment. Furthermore, the Google Earth Engine cloud-based platform^113^ provides geospatial data on a variety of high-impact societal issues on planetary-scale. The exploitation of such platforms, coupled with the application of tailored data analysis tools and models and experimental evidence supported by statistical analysis, lay the foundation for the achievement of high-level multidisciplinary characterizations indispensable for the achievement of the sustainable use of marine and coastal resources, as also required by the Unite Nations Sustainable Development Goals.

## Proposal of a coastal management matrix

By considering the set of surveys and analysis suggested as Stage 1 and Stage 2 (cf. sections “Activities envisaged for the definition of the geological s.l.model” and “Activities envisaged for the definition of the anthropogenic impact on marine and coastal sediments.”), it is possible to define the overall level of contamination for the marine sediments, by both accounting for the pollutants concentrations and relating their spatial distribution with the main site-specific geological, sedimentological, and stratigraphic characteristics. Furthermore, accounting for the evaluation of the expected variation in coastal forcing agents as consequences of both weather- and anthropogenic-related modifications (Stage 3, cf. section “Activities envisaged for the definition of expected morphodynamic variations.”), it is possible to estimate local variations in physical processes and their influence on the potential redistribution of contaminants.

**Figure 9:**
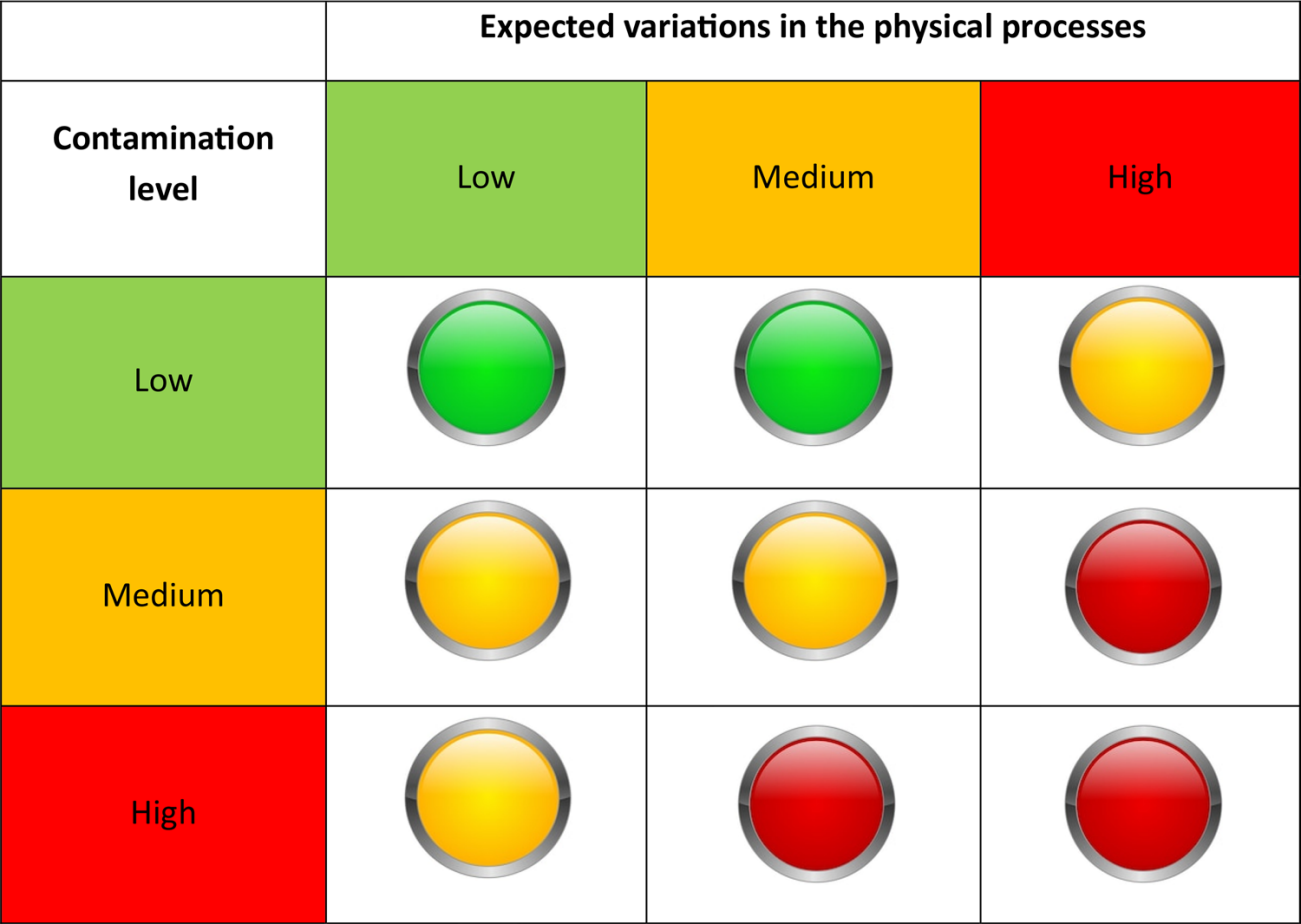
The “traffic light” matrix proposed for the definition of tailored management activities to be performed in highly contaminated coastal areas.

The combined analysis of these parameters allowed us to propose an evaluation matrix as support for the selection of practices and actions to be undertaken for the management of highly contaminated coastal sites. For this purpose, a traffic light structure is here adopted (Fig. 9). The structure is inspired by a classical risk matrix, in which parameters and/or indices are combined to determine an overall risk level for sustainable coastal planning and management^114–116^.

Specifically, the traffic light is built by considering three levels for the contamination parameter:

- Low contamination level: the concentration of chemical pollutants is higher than the local background values but lower than national/site-specific action value limits;
- Medium contamination level: the concentration of chemical pollutants is higher than national/site-specific action value limits, but the contamination only affects the uppermost sediment layer (0–0.50 m);
- High contamination level: the concentration of chemical pollutants is higher than national/site-specific action value limits and the contamination affects both surficial and sub-surficial sediment layers.

Furthermore, the potential impacts induced by expected variations in physical processes (expressed in terms of morphological and dynamic modifications affecting both the continental and the marine areas) are classified as follows:

- Low: expected coastal modifications are limited and the coastal site is considered able to cope with their potential impact since coastal sedimentary processes prevail; sea-bottom sediments are not expected to be affected by erosion and dispersion processes;
- Medium: expected coastal modifications are relevant but the site is characterized by morpho-dynamic responses that allow the coastal environmental adaptation; sea-bottom sediments are expected to be moved;
- High: expected coastal modifications are very strong and the coastal site is considered unable to cope with their potential impacts since no morphodynamic responses are expected; sea-bottom sediments are expected to be affected by erosion and dispersion processes.

By combining the above-mentioned parameters, the following conditions can be obtained:

(i) The “green light” case, which is obtained when the contamination level can be considered low and the morphodynamic variations are classified as low or medium. In this case, tailored monitoring programs are required. The monitoring programs aim to ensure the protection of coastal ecosystems and to keep the health and environmental risk at low levels;
(ii) The “yellow light” case, which is obtained when both the contamination level and the expected morphodynamic variations are classified as medium or high. In this case, safety actions need to be implemented in order to reduce the sediment mobility and the dispersion of the contaminants. Such kinds of actions may include both the definition of interdiction zones and ecosystem restoration.
(iii) The “red light” case, which is obtained when the contamination level is high and the expected morphodynamic variations are medium or high. In this case, high reclamation activities, both in-situ and ex-situ, are required to reduce the contamination. Furthermore, management plans are required to reduce health risks and avoid ecosystem losses.

## Conclusion

In this research, a comprehensive overview of surveys and analyses potentially designable for the integrated characterization of highly contaminated coastal sites is presented. In detail, three different phases of investigation are proposed as reference guidelines. The proposed investigations allow to obtain a set of information that meets the requirements of the main European Directives focused on the maritime spatial planning and marine environmental policy.

In order to provide the scientific baseline of the geological, geomorphological, and sedimentological setting, activities described in the first phase are aimed at supporting the definition of the site-specific geological s.l. model of the investigated marine sector.

To meet the requirements of the MSFD for the achievement of a good ecological status of the marine environments, activities proposed in the second phase are focused on the evaluation of the anthropogenic impacts on marine matrices (surface and sub-surface sediments), in terms of presence of pollutants and human footprint on the seafloor, which are both indicated as main pressures and impacts in Annex III of the MSFD.

Furthermore, surveys illustrated in the third phase support the evaluation of the expected morpho-dynamic variations in physical coastal processes induced by climate change and anthropogenic activities so to increase the analysis of potential coastal resilience and support the sustainable future coastal development (as required in Art. 5 of MSP Directive).

The research was also focused on the comprehensive analysis of the surveys performed to characterize the highly contaminated coastal sites located in the Apulia region. This analysis highlights how geological surveys may be exploited to obtain data and information suitable for both the definition of the site-specific geological model and the analysis of the 3D distribution of contaminants in marine sediments. This last aspect places importance on the need for multidisciplinary evaluations.

Finally, a very simple matrix is proposed to combine environmental quality analysis with the evaluation of future variations in morphodynamic processes. The synthesis of the procedures proposed in this study is provided in the form of infographics to facilitate their diffusion and sharing at both the scientific, policy, and administrative levels (cf. Appendix 1, 2, 3, 4).

In conclusion, the outcomes of this research represent the foundation of the investigation procedure to be used as a reference for the characterization of regional and national highly contaminated coastal sites. In accordance with the international data sharing principles, each investigation phase is also presented as infographics to be used as informative material for the stakeholder’s engagement as well as for the dissemination of the research project outcomes.

### Supplementary Information

Supplementary Information.

## Data Availability

All data are available on request to the corresponding author.
