# Supplementary material for: A set of guidelines as support for the integrated geo-environmental characterization of highly contaminated coastal sites
Source: Sci Rep. 2024 Apr 8;14:8198. doi: 10.1038/s41598-024-58686-4 (PMC11001938; doi:10.1038/s41598-024-58686-4)
Supplement: Supplementary file 1 — Supplementary Information. [file 41598_2024_58686_MOESM1_ESM.pdf]

APPENDIX TO THE MANUSCRIPT

A set of guidelines as support for the integrated geo-environmental characterization of highly contaminated coastal sites.

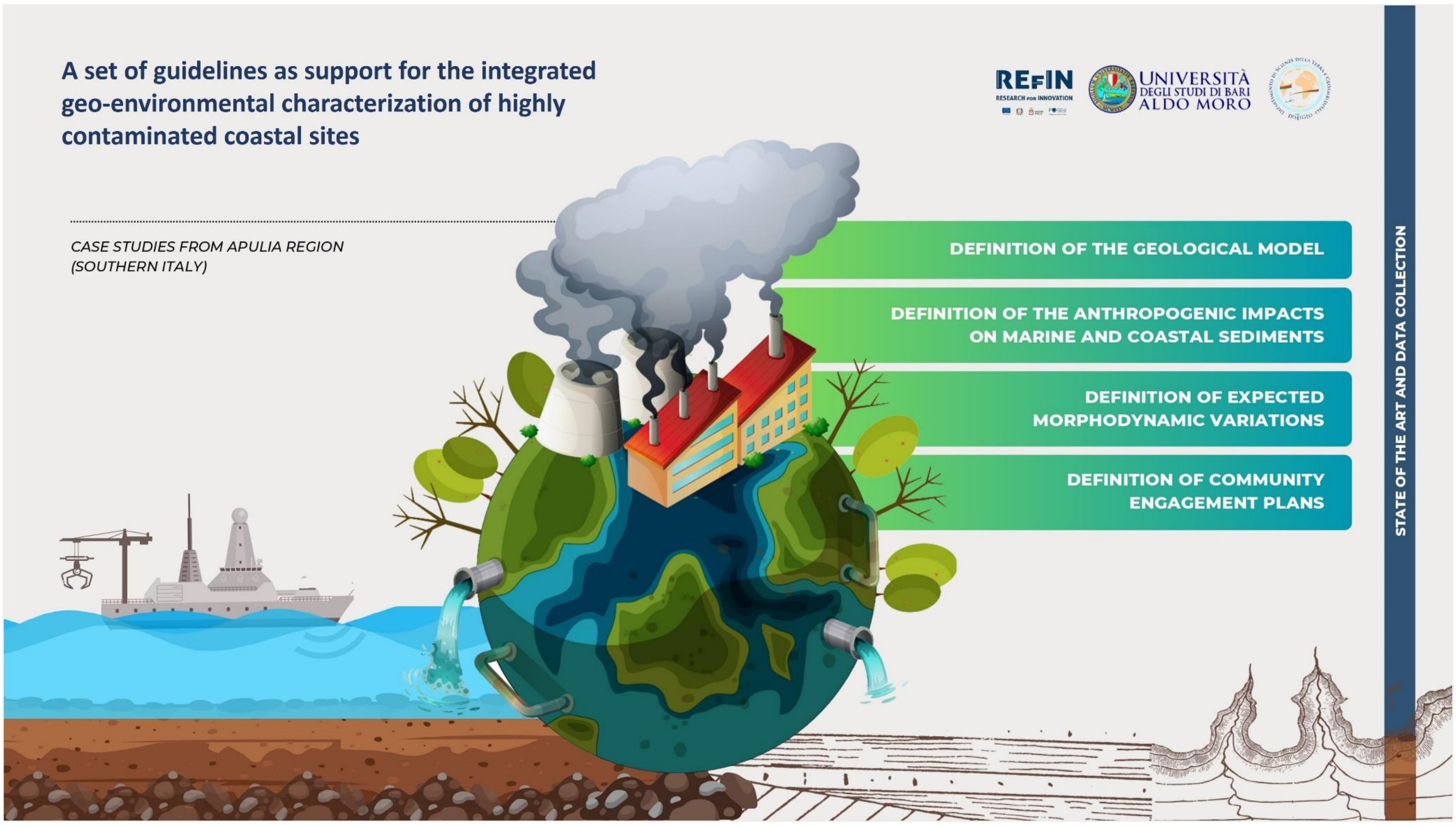

Graphical project for clipart images and infographics provided as Appendix to the manuscript was designed by Giovanni Pellegrino, Visual Designer and Brand Strategist (source images: canva.com).

REFIN RESEARCH FOR INNOVATION

UNIVERSITÀ DEGLI STUDI DI BARI ALDO MORO

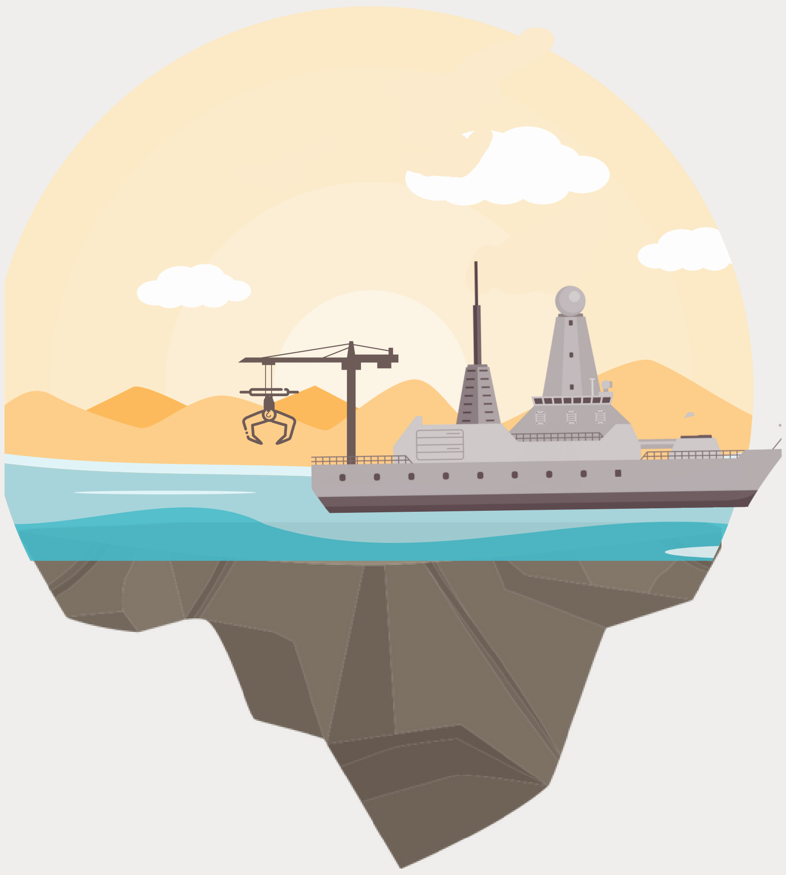

DEFINITION OF THE GEOLOGICAL MODEL

The site-specific geological model is used for the analysis of the different sedimentary units, the definition of their geometric relationships, the evaluation of the thickness of superficial deposits, and the spatial distribution of each sediment unit.
Indirect geophysical surveys (seismic, acoustic, and geoelectric) allow to define over a wide area the main geological structures and their geometries within the stratigraphic succession.
Direct analyses (sedimentological, geochemical, mineralogical, and biogeochemical) allow to define the lithostratigraphic characteristics of rock outcrops and marine substrate as well as the inorganic characteristics of sediments.

ACQUISITION OF GEOLOGICAL DATA DERIVED FROM PREVIOUS CHARACTERIZATION ACTIVITIES, GEOPHYSICAL SURVEYS, AND ANALYSES. IN SITU GEOMORPHOLOGICAL SURVEYS AND CORE SAMPLING.

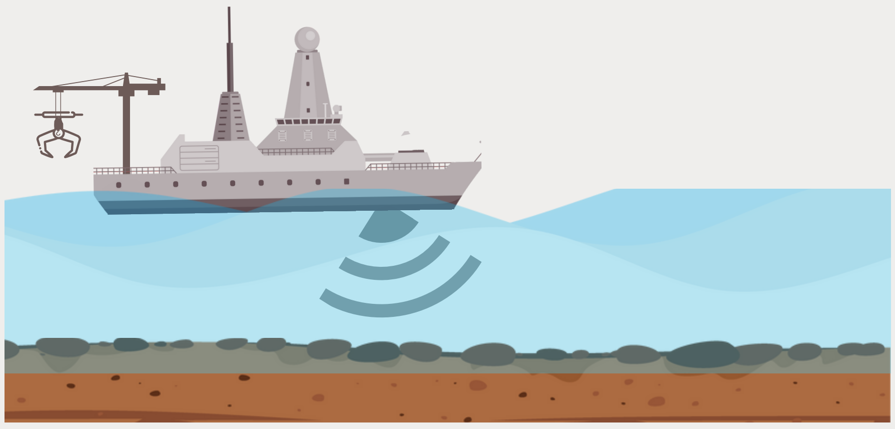

SEISMIC AND ACOUSTIC SURVEYS

Seismic reflection investigations are used in subsurface exploration to recognise the stratigraphic and structure of geological units. Marine acoustic investigations are used to obtain high-resolution morpho-bathymetric information.

- HIGH-RESOLUTION SINGLE-CHANNEL REFLECTION SEISMIC SURVEYS
  1. SPARKER
  2. SUB BOTTOM PROFILE
- HIGH-RESOLUTION MULTI-CHANNEL REFLECTION SEISMIC SURVEYS
- SINGLE/MULTI BEAM ECHO SOUNDER
- SIDE SCAN SONAR

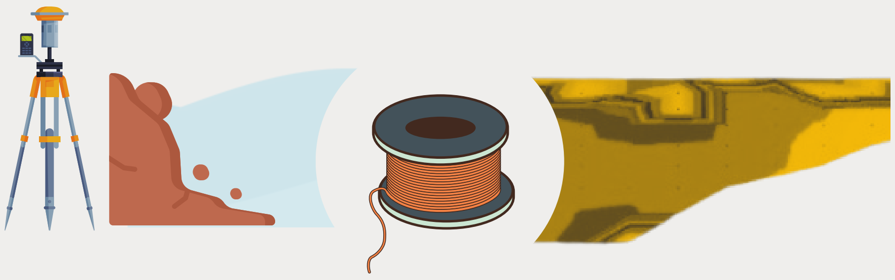

GEOELECTRIC AND HYDROGEOLOGICAL SURVEYS

Geoelectric investigation (marine and terrestrial) allows to define the electrical resistivity of rocks and water bodies and, therefore, the main lithological discontinuities and the fresh-saltwater interface.

- VERTICAL ELECTRICAL SOUNDING (VES)
- ELECTRICAL RESISTIVITY TOMOGRAPHY (ERT)

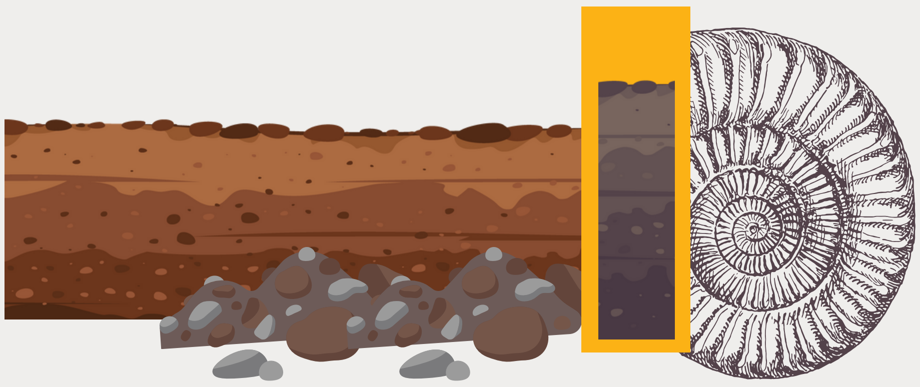

SEDIMENTOLOGICAL ANALYSIS

Sedimentological analyses allow to define stratigraphic, radiographic, and radiometric characteristics of the marine and coastal sampled sediments and to correlate seismic interpretations.

- VISUAL DESCRIPTION OF THE SAMPLES
- GRANULOMETRIC ANALYSIS
- MAGNETIC ANALYSIS
- RADIOGRAPHIC ANALYSIS

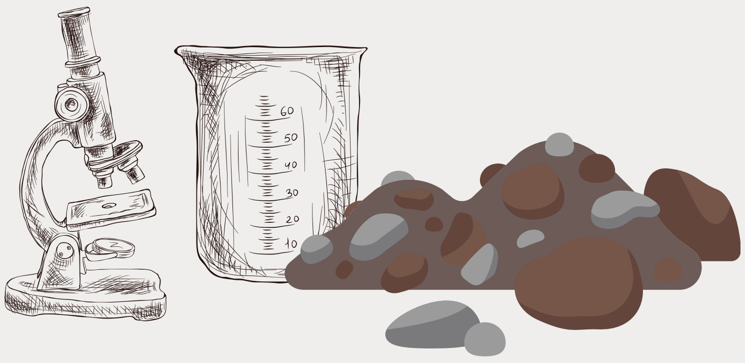

GEOCHEMICAL, MINERALOGICAL, AND BIOGEOCHEMICAL ANALYSES

Direct geochemical and mineralogical analyses are performed to characterize the current and sub-actual sediments from a mineralogical and inorganic component perspective. Sample dating and bio-geochemical analyses allow to reconstruct the paleo-environmental conditions.

- X RAY FLUORESCENCE–XRF
- X RAY POWDER DIFFRACTION–XRPD
- TRANSMISSION ELECTRON MICROSCOPY (TEM)
- SCANNING ELECTRON MICROSCOPY (SEM)
- RADIOCARBON DATING-14C
- PALYNOLOGICAL ANALYSIS

DEFINITION OF THE ANTHROPOGENIC IMPACTS ON MARINE AND COASTAL SEDIMENTS

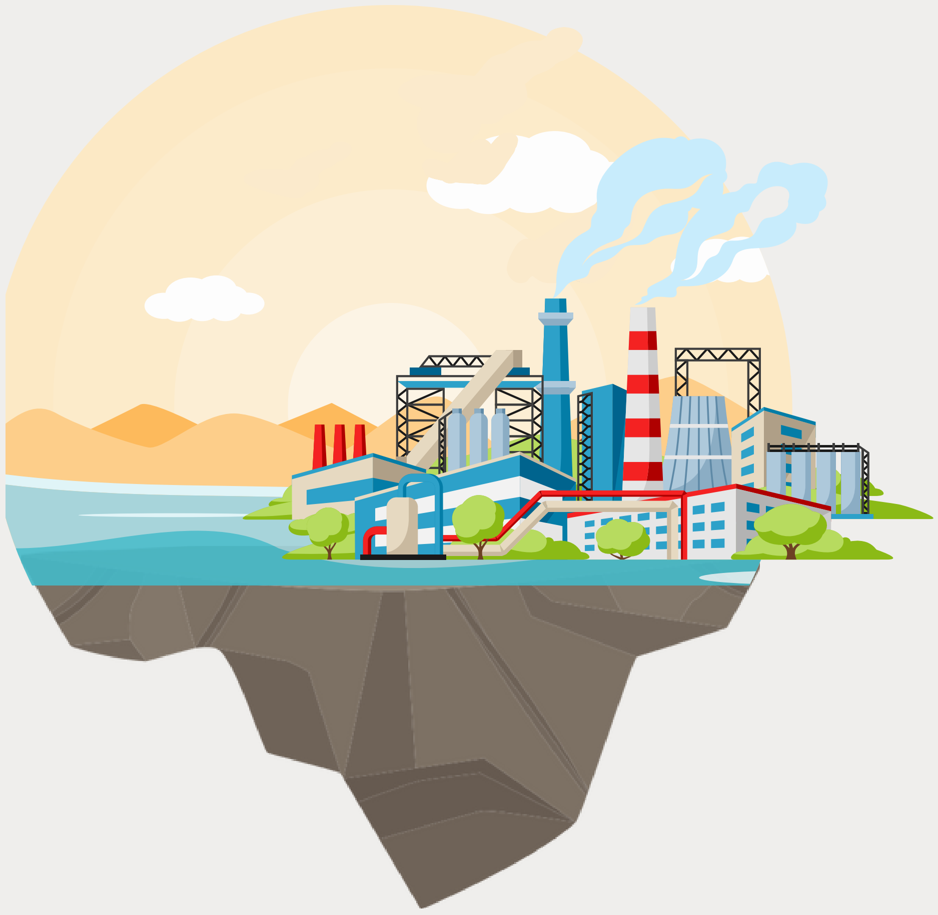

Marine and coastal sediments in highly contaminated coastal sites can be directly and indirectly impacted by anthropogenic activities. Direct impacts can be defined through geochemical analyses to estimate concentrations of organic and inorganic pollutants (including microplastics) in surface and sub-surface sediments. Indirect impacts, which include anthropogenic footprints on seabed morphology, i.e., the impact of maritime activities carried out in coastal areas (as shipping, fishing, and shellfish farming) and the dumping of litter and mega-litter, can be assessed through the interpretation of high-resolution geophysical data (i.e., acoustic and magnetometric surveys).

DESCRIPTION OF HISTORICAL AND CURRENT ANTHROPOGENIC ACTIVITIES CARRIED OUT IN THE SELECTED SIN. COLLECTION OF POLLUTION DATA FROM PREVIOUS CHARACTERIZATION PLANS. SEDIMENT SAMPLING.

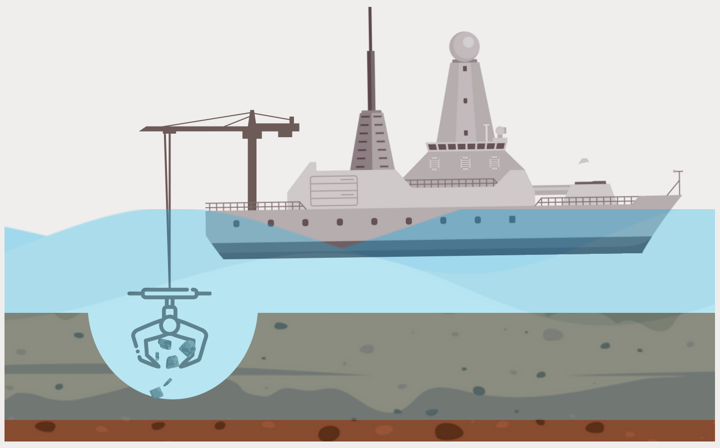

SEDIMENT SAMPLING

The identification of suitable sites for sediment sampling and the definition of the length of sediment cores to be taken are supported by information obtained from preliminary seismic surveys.

- GRAB SAMPLING (SURFICIAL SEDIMENTS)
- CORE SAMPLING (SURFICIAL, SUB-SURFICIAL, AND DEEP SEDIMENTS)

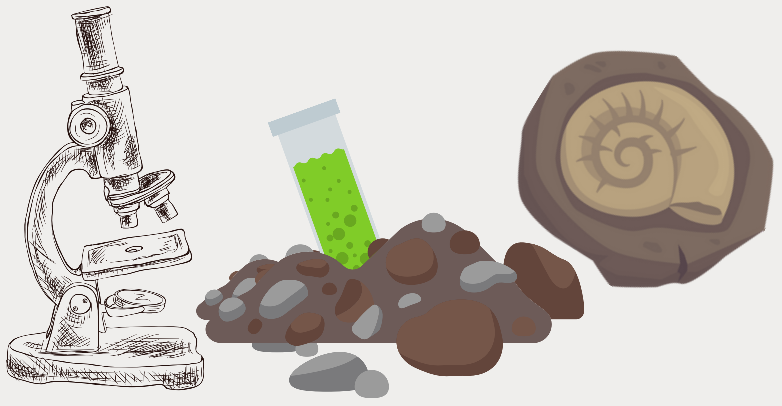

GEOCHEMICAL ANALYSES

The assessment of pollutant concentrations in marine and coastal sediments and their comparison with limit values and sediment quality guidelines allows the level of contamination and related risk to be estimated. In addition, site-specific background values can be defined from the analysis of deeper sediment layers.

- INDUCTIVELY COUPLED PLASMA MASS SPECTROMETRY (ICP-MS)
- GAS CHROMATOGRAPHY MASS SPECTROMETRY (GC-MS; GC/MS-MS)
- FOURIER TRANSFORM INFRARED SPECTROSCOPY (FT-IR)
- RAMAN SPECTROSCOPY
- LASER INDUCED BREAKDOWN SPECTROSCOPY (LIBS)
- HYPERSPECTRAL IMAGING (HSI)
- ORGANIC CARBON CONTENT ANALYSIS

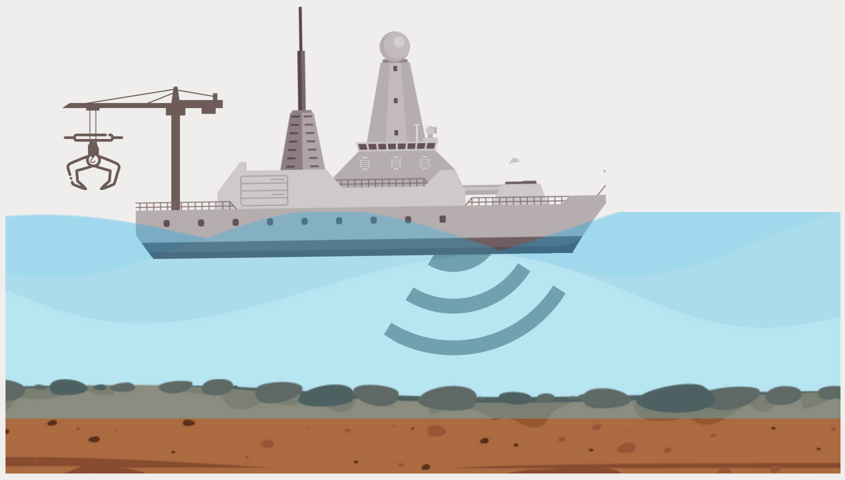

GEOPHYSICAL SURVEYS

The interpretation of high-resolution morpho-bathymetric acoustic data allows the identification on the seabed of imprints and traces of anthropogenic activities. The acquisition of magnetometric data support the detection of buried structures and objects.

- SINGLE/MULTI BEAM ECHO SOUNDER SURVEYS
- SIDE SCAN SONAR SURVEYS
- MAGNETOMETRIC SURVEYS

REFIN
RESEARCH FOR INNOVATION

UNIVERSITÀ DEGLI STUDI DI BARI ALDO MORO

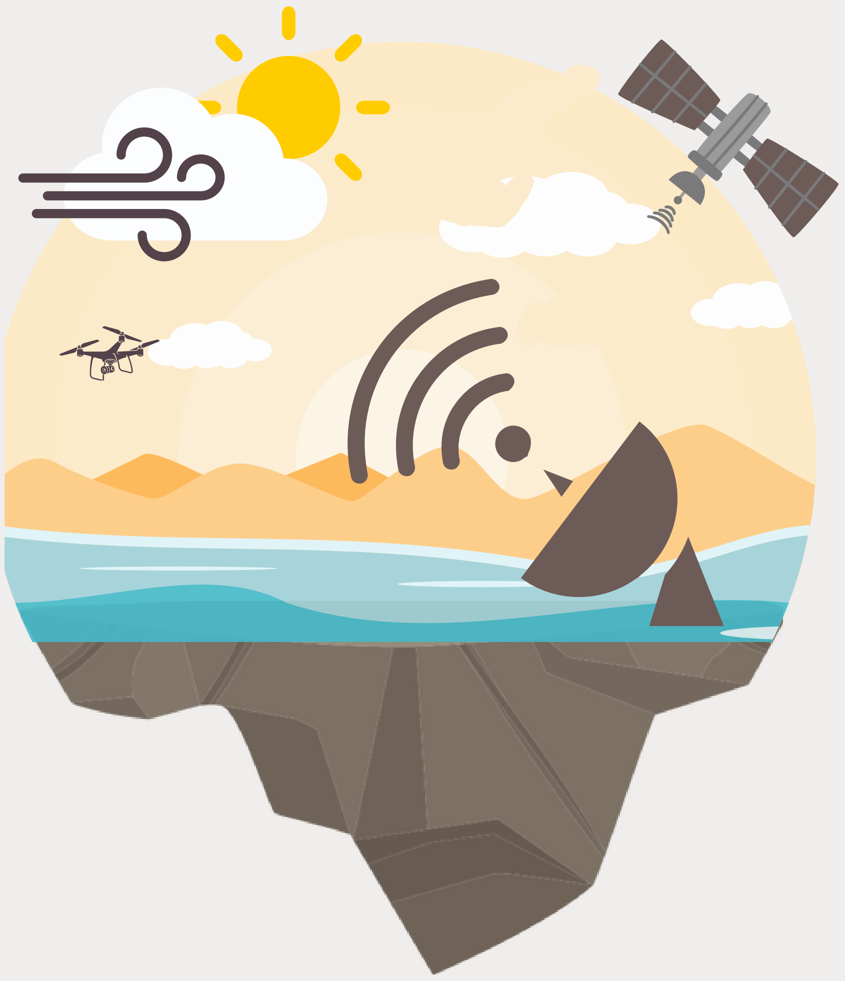

DEFINITION OF THE EXPECTED MORPHODYNAMIC VARIATIONS

Ongoing climate-related changes in marine and atmospheric variables are expected to induce cascading effects on coastal physical processes (e.g., wave climate, current-sediment interactions, storm surges, erosion processes). Similarly, future coastal anthropogenic interventions may cause changes in longshore sediment transport. The alteration of energy and mass balances generated by these processes may trigger contaminant mobilization mechanisms therefore the identification of geomorphodynamic interactions with the contamination features supports the site-specific risk assessment procedure.

COLLECTION OF BATHYMETRIC AND TOPOGRAPHIC DATA ALREADY AVAILABLE AT THE NATIONAL AND REGIONAL SCALE FOR THE INVESTIGATED COASTAL SITES.

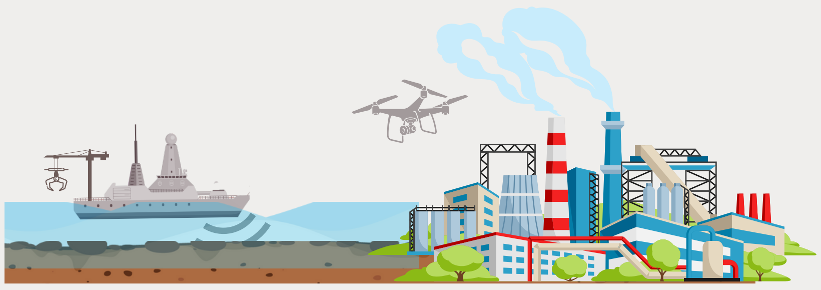

HIGH-RESOLUTION TOPOGRAPHIC & BATHYMETRIC SURVEYS

Innovative technologies allow to obtain high resolution bathymetric and topographic coastal models and to define submerged and emerged morphologies with high vertical accuracy and to assess their variation over time.

- UNMANNED AERIAL VEHICLES (UAVS) SURVEYS
- TERRESTRIAL LASER SCANNER (TLS) SURVEYS
- OPTICAL TOTAL STATION SURVEYS
- SINGLE/MULTI BEAM ECHO SOUNDER

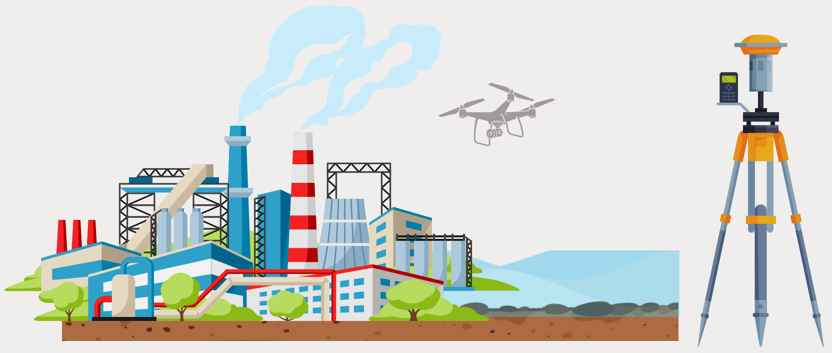

MEDIUM AND SHORT-TERM SHORELINE OBSERVATIONS

The analysis of current shoreline displacements allows for an indirect assessment of the predominant site-specific morphodynamic conditions and thus the identification of coastal erosional focuses along the investigated SIN. The medium-term analysis is also used to obtain projections of future shoreline position.

- UNMANNED AERIAL VEHICLES (UAVS) SURVEYS
- DIFFERENTIAL GPS SURVEYS

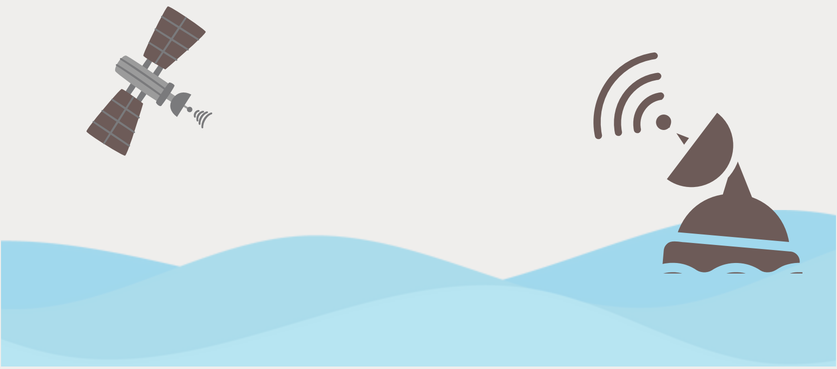

ANALYSIS OF ONGOING VARIATIONS IN MARINE PARAMETERS

The evaluation of future variations in marine parameters (i.e., currents, waves, relative sea level, etc.), generally expressed in terms of anomalies, is performed by comparing historical data with projections obtained from very high-resolution climate models.

- ACQUISITION OF HISTORICAL DATA FOR MARINE VARIABLES FROM NATIONAL, REGIONAL, AND LOCAL REPOSITORIES
- INSTALLATION OF IN-SITU INSTRUMENTS

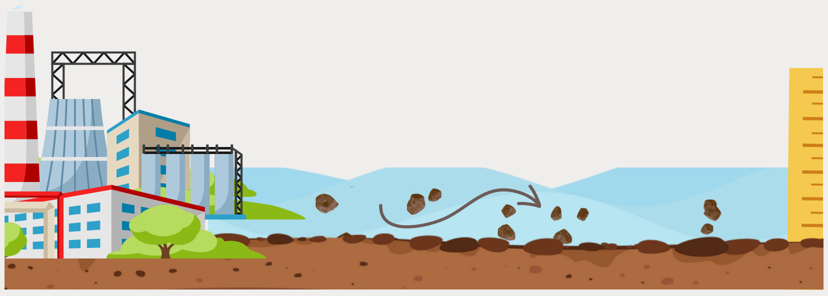

ANALYSIS OF FUTURE VARIATIONS IN MARINE PARAMETERS

The evaluation of the current variations in marine parameters (i.e., currents, waves, relative sea level) is performed by analysing historical data and current local observations obtained from the installation of monitoring instruments along the coastline.

- ACQUISITION OF DATA PROJECTIONS FOR MARINE VARIABLES FROM REFERENCES DATA PROVIDERS SUCH AS COPERNICUS PLATFORMS AND IPCC REPORTS AND PLATFORMS.
- SET UP OF HIGH-PERFORMANCE MATHEMATICAL MODELS

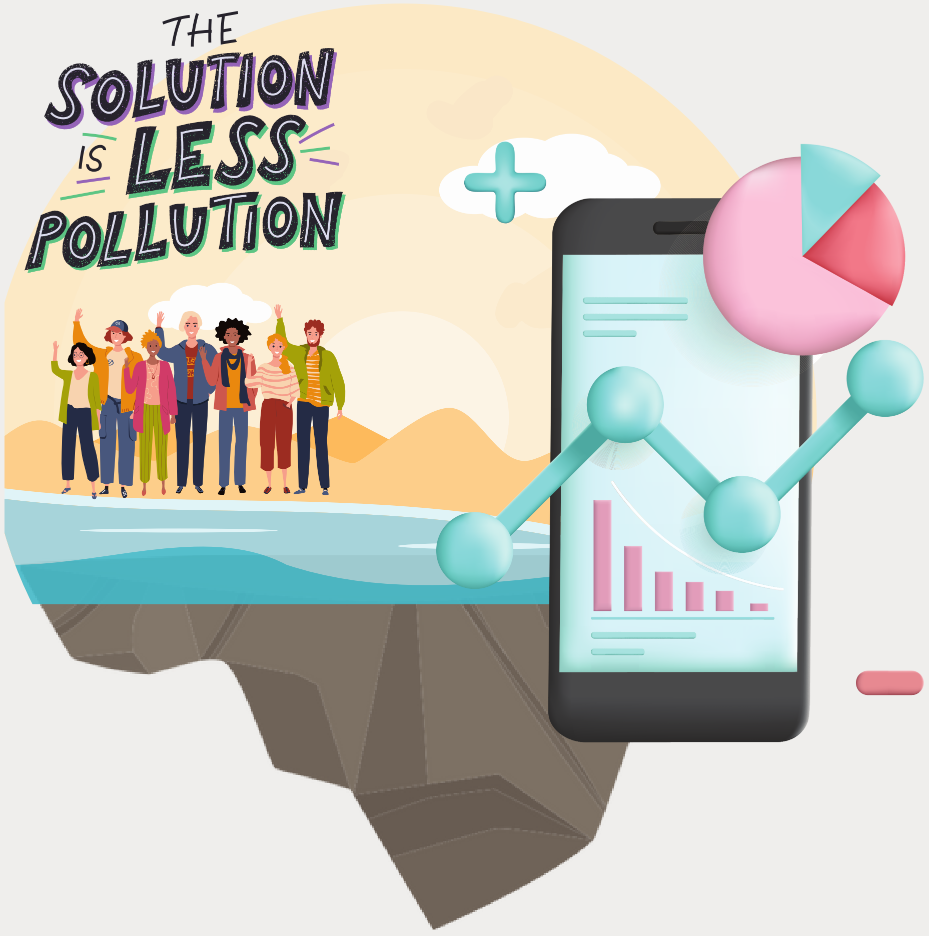

DEFINITION OF COMMUNITY ENGAGEMENT PLANS

A participatory stakeholder process lays the foundations for extensive citizens' participation in public processes resulting in the identification of collectively accepted site-specific solutions for the management of highly contaminated coastal sites. Stakeholders' engagement in participatory decision-making at the local and regional scale is fundamental for the definition of actions aimed at the reduction of environmental and health risks. Furthermore, citizens can become active participants in the knowledge process by supporting the in-situ and real-time acquisition of information.

IDENTIFICATION AND ELICITATION OF STAKEHOLDERS FROM POTENTIALLY INTERESTED GROUPS TO ANY LEVEL OF SOCIETY. ANALYSIS OF THE ALREADY AVAILABLE CITIZEN SCIENCE PROGRAMS.

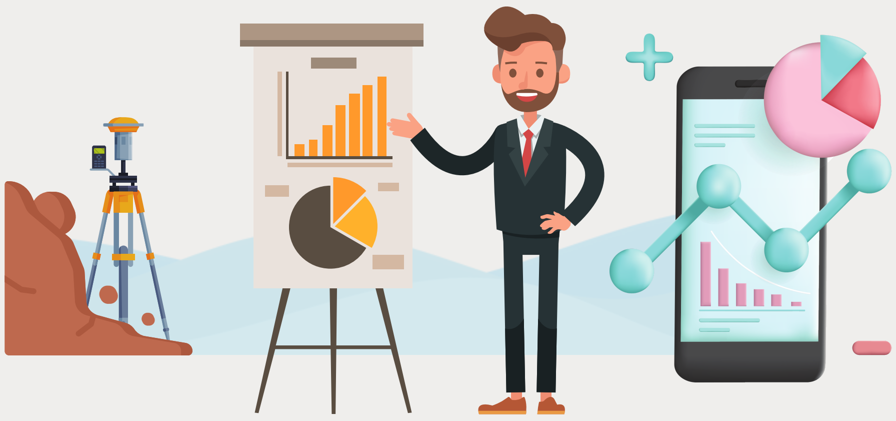

DISSEMINATION OF THE RESULTS OBTAINED FROM THE DATA ANALYSIS ACTIVITIES.

- DEVELOPMENT OF USER-FRIENDLY WEBGIS PLATFORM
- DEVELOPMENT OF ILLUSTRATIVE VOLUMES
- ORGANIZATION OF MEETING AND WORKSHOP EVENTS

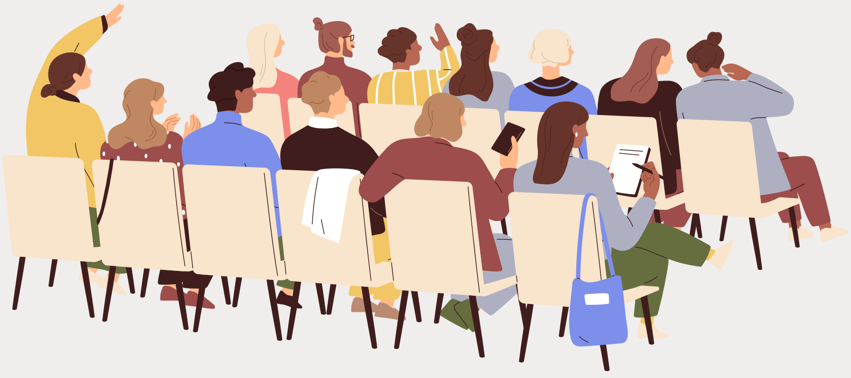

CITIZEN ENGAGEMENT OF CITIZENS IN THE KNOWLEDGE PROCESS.

- ORGANIZATION OF EVENTS AIMED AT INCREASING THE ENVIRONMENTAL SENSITIVITY OF THE CITIZENS INVOLVED IN FIELD DATA COLLECTION
- DEVELOPMENT OF SPECIFIC MOBILE APPLICATIONS FOR DATA COLLECTION

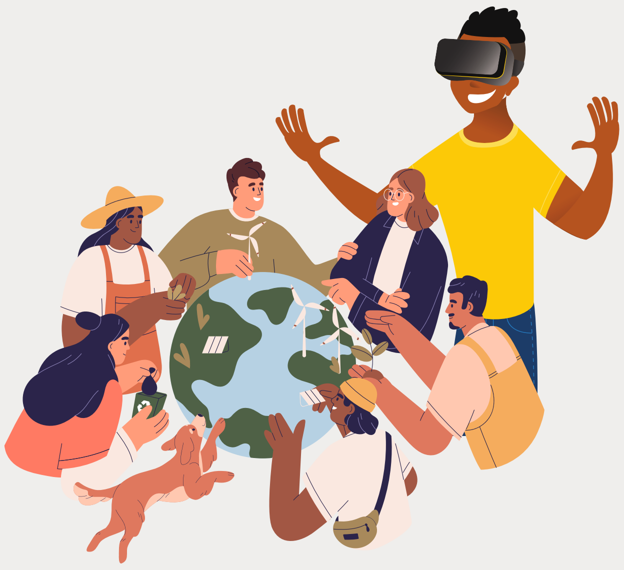

CITIZEN ENGAGEMENT IN THE DECISION-MAKING PROCESS.

- DEVELOPMENT OF A "MANAGEMENT PRACTICES" DATABASE TO CONSULT TAILORED REMEDIATION ACTIONS
- DEVELOPMENT OF A TAILORED DECISION SUPPORT TOOL FOR THE IDENTIFICATION OF THE MOST ACCEPTABLE STRATEGIES
- DEVELOPMENT OF VR-BASED VISUALIZATION AND ANALYSIS TOOLS TO HELP USERS IN THE VISUALIZATION OF COMPLEX DATASETS AND TO SUPPORT THEIR ANALYSIS AND UNDERSTANDING
